# Clinical cell-surface targets in metastatic and primary solid cancers

**DOI:** 10.1172/jci.insight.183674

**Published:** 2024-09-24

**Authors:** Marina N. Sharifi, Yue Shi, Matthew R. Chrostek, S. Carson Callahan, Tianfu Shang, Tracy J. Berg, Kyle T. Helzer, Matthew L. Bootsma, Martin Sjöström, Andreas Josefsson, Felix Y. Feng, Laura B. Huffman, Chris Schulte, Grace C. Blitzer, Quaovi H. Sodji, Zachary S. Morris, Vincent T. Ma, Labros Meimetis, David Kosoff, Amy K. Taylor, Aaron M. LeBeau, Joshua M. Lang, Shuang G. Zhao

**Affiliations:** 1 Department of Medicine,; 2 Carbone Cancer Center, and; 3 Department of Human Oncology, University of Wisconsin, Madison, Wisconsin, USA.; 4 Department of Clinical Sciences Lund, Division of Oncology, Lund University, Lund, Sweden.; 5 Department of Hematology, Oncology and Radiation Physics, Skåne University Hospital, Lund, Sweden.; 6 Wallenberg Center for Molecular Medicine, Urology, Department of Diagnostics and Intervention, Umeå University, Umea, Sweden.; 7 Departments of Radiation Oncology, Urology, and Medicine, UCSF, San Francisco, California, USA.; 8 Department of Obstetrics and Gynecology and; 9 Department of Radiology, University of Wisconsin, Madison, Wisconsin, USA.; 10 William S. Middleton Memorial Veterans’ Hospital, Madison, Wisconsin, USA.; 11 Department of Pathology and Laboratory Medicine, University of Wisconsin, Madison, Wisconsin, USA.

**Keywords:** Oncology, Clinical practice

## Abstract

Therapies against cell-surface targets (CSTs) represent an emerging treatment class in solid malignancies. However, high-throughput investigations of CST expression across cancer types have been reliant on data sets of mostly primary tumors, despite therapeutic use most commonly in metastatic disease. We identified a total of 818 clinical trials of CST therapies with 78 CSTs. We assembled a data set spanning RNA-seq and microarrays in 7,927 benign samples, 16,866 primary tumor samples, and 6,124 metastatic tumor samples. We also utilized single-cell RNA-seq data from 36 benign tissues and 558 primary and metastatic tumor samples, and matched RNA versus protein expression in 29 benign tissue samples, 1,075 tumor samples, and 942 cell lines. High RNA expression accurately predicted high protein expression across CST therapies in benign tissues, tumor samples, and cell lines. We compared metastatic versus primary tumor expression, identified potential opportunities for repositioning, and matched cell lines to tumor types based on CST and global RNA expression. We evaluated single-cell heterogeneity across tumors, and identified rare normal cell subpopulations that may contribute to toxicity. Finally, we identified combinations of CST therapies for which bispecific approaches could improve tumor specificity. This study helps better define the landscape of CST expression in metastatic and primary cancers.

## Abstract 2

Global analysis of cell surface target expression in primary vs. metastasis, cell line matching, single-cell heterogeneity, and combinatorial bi-specific approaches.

## Introduction

Cell-surface-targeted therapies have emerged as a highly effective new class of therapies for both hematologic and solid tumor malignancies. Unlike conventional cytotoxic chemotherapy, therapies targeting cell-surface proteins offer a strategy to increase cancer cell specificity while minimizing toxicities. Multiple types of cell-surface protein–targeted therapies have been developed with different mechanisms of action. Antibody-drug conjugates (ADCs), which selectively deliver cytotoxic payloads to tumor cells via antibodies against tumor cell–surface targets, represent a highly successful class with multiple FDA approvals. Several other classes of cell-surface-targeted therapies have received FDA approval, including radiopharmaceutical therapies (RPTs), which deliver a therapeutic radionuclide, as well as a number of immunomodulatory cell-surface-targeted therapies, including chimeric antigen receptor T cells (CAR-Ts), CAR natural killer cells (CAR-NKs), CAR dendritic cells (CAR-DCs), CAR macrophages (CAR-Ms), and bispecific T cell engagers (BiTEs).

In solid tumors, there are 10 currently FDA-approved cell-surface-targeted therapies. ADCs are the most common class, with T-DM1 (targeting HER2) approved in HER2^+^ breast cancer, fam-trastuzumab deruxtecan (targeting HER2) approved for HER2^+^ solid cancers and HER2-mutant non–small cell lung cancer (NSCLC), enfortumab vedotin (targeting NECTIN4) approved in bladder cancer, sacituzumab govitecan (targeting TROP2) approved for HER2-negative breast cancer and bladder cancer, mirvetuximab soravtansine (targeting FOLR1) approved for FOLR1-positive platinum-resistant epithelial ovarian, fallopian tube, and peritoneal cancers, and tisotumab vedotin (targeting tissue factor) approved for cervical cancer. RPTs are the next most common, with ^177^Lu-PSMA-617 (targeting PSMA) approved for prostate cancer and ^177^Lu-DOTATATE (targeting SSTR2) approved for somatostatin receptor–positive gastroenteropancreatic neuroendocrine tumors. Finally, tebentafusp-tebn is a BiTE (targeting gp100) approved for uveal melanoma and is in trials for cutaneous melanoma. Tarlatamab, a DLL3-targeted BiTE, was also approved recently for small cell lung cancer (SCLC). Dozens of other cell-surface-targeted therapies for these and other proteins are in clinical trials. This class of agents represents an important emerging addition to our antineoplastic armamentarium. As the potential options multiply, a method to prioritize between a multitude of potential targets is needed.

To date, the identification of cell-surface markers for new therapeutic targets beyond well-established tumor cell-surface proteins such as HER2 and PSMA has utilized data from large sequencing data sets such as The Cancer Genome Atlas (TCGA). These data are primarily obtained from nonmetastatic tumor tissue samples, owing to the challenges of profiling large metastatic tissue data sets. However, tumor expression profiles have been shown to differ in metastatic disease (1), suggesting a gap in knowledge of optimal cell-surface targets in the metastatic setting. To address this, we curated a list of cell-surface targets with agents currently under study in clinical trials. We established in paired protein-RNA data sets that high gene expression was strongly associated with high protein expression of these targets. We then evaluated these targets in a large integrated RNA expression data set of metastatic tumors paired with primary tumors to understand patterns of cell-surface target expression between and within tumor types. We further integrated normal tissue RNA-seq as well as normal and tumor tissue single-cell RNA-seq (scRNA-seq) data sets into our analysis to understand the contribution of normal tissue expression and single-cell heterogeneity to potential efficacy and toxicity profiles across these cell-surface targets.

## Results

### Overview of cell-surface targets in clinical trials/practice.

We first performed a comprehensive search of ClinicalTrials.gov to identify all interventional clinical trials (as of October 31, 2023) of surface-targeted cancer therapies for adult solid tumors. We identified a total of 818 trials (Supplemental Table 1; supplemental material available online with this article; https://doi.org/10.1172/jci.insight.183674DS1). We manually curated the cell-surface protein targets, inclusion criteria, and phase of each trial based on information from trial details and related publications. A total of 78 cell-surface protein targets were identified, including 9 targets with FDA-approved therapies for solid tumors (Supplemental Figure 1A). Of these trials, the majority (65%) assessed ADCs (Supplemental Figure 1B), and 82% were early phase (I–II) trials. Given that cell-surface-targeted therapies represent an emerging modality, most of the clinical trials were currently active at the time of review (Supplemental Figure 1C).

### High-throughput data sets.

**Figure 1:**
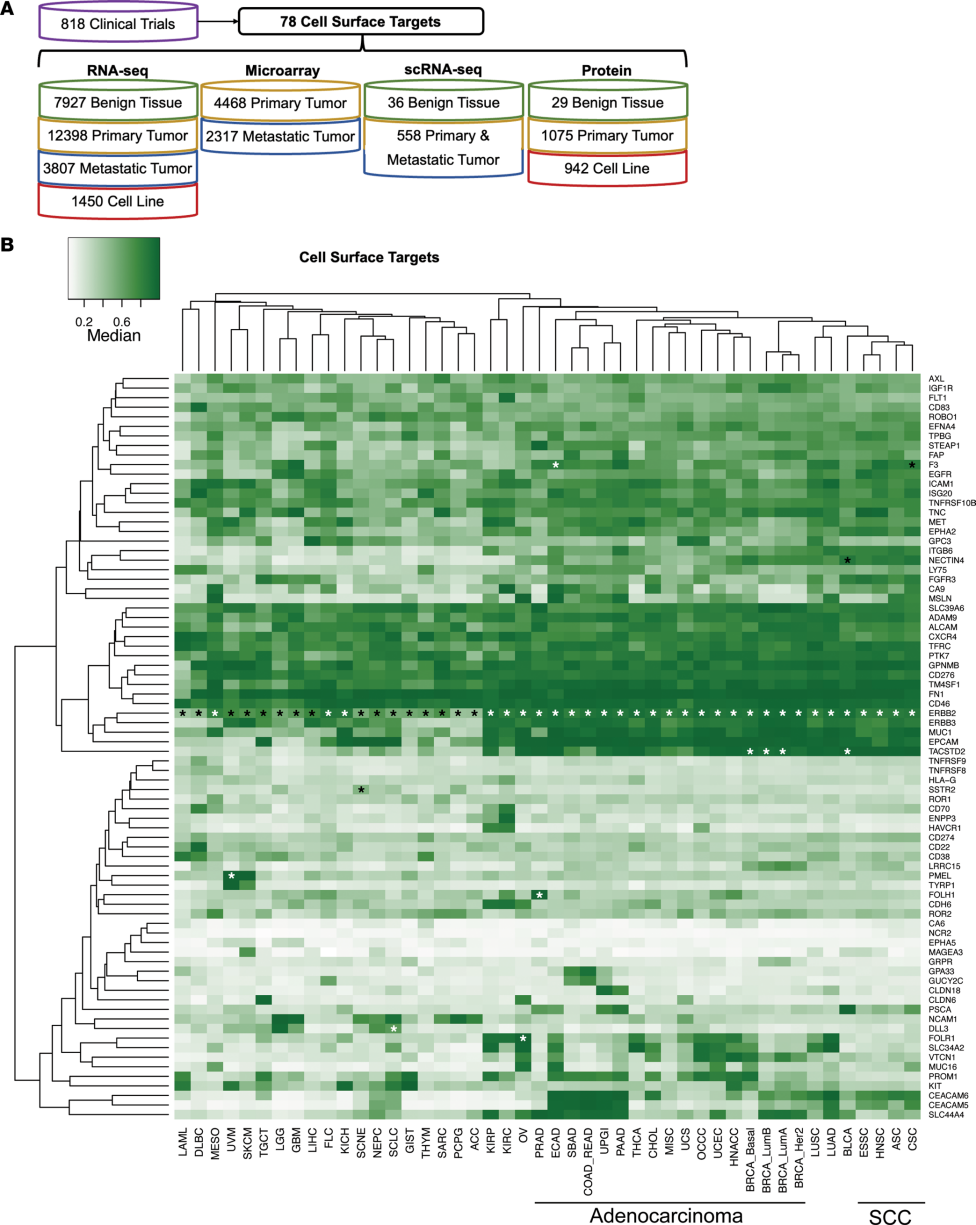
Cell-surface target tumor expression. (**A**) Schematic of all data used in this study. (**B**) Hierarchical clustering of median bulk RNA expression of 78 cell surface protein targets (rows) across 43 solid cancer types (columns: primary and metastatic). RNA expression levels are percentile rank normalized across all genes, ranging from 0 to 1. Black and white asterisks = FDA-approved indication.

To best understand how cell-surface target expression varies across cancer types, benign tissues, and cell lines, we assembled a large data set spanning RNA-seq data in 7,927 benign samples, 12,398 primary tumor samples (mostly from TCGA), and 1,450 cell lines (Figure 1A). Additionally, we also included 3,807 metastatic tumor samples (Supplemental Figure 2). In addition to RNA-seq, we also included RNA expression from 4,468 primary tumor samples and 2,317 metastatic tumor samples profiled with gene expression microarrays (Supplemental Figure 3). To facilitate comparisons between disparate data sets, we normalized each gene to the percentile expression within each sample. We also utilized scRNA-seq data from 36 benign tissues and 558 primary and metastatic tumor samples. Finally, we also compared matched RNA versus protein expression in 29 benign tissue samples, 1,075 tumor samples, and 942 cell lines. In aggregate, these data allow us to comprehensively explore cell-surface target expression across solid tumors.

### High cell-surface target RNA expression accurately predicts high protein expression.

We first examined cell-surface RNA versus protein expression in 3 data sets of cancer tissue samples, cell lines, and benign tissues with matched RNA-seq and mass spectrometry. For cell-surface expression, our focus was on how accurately high RNA expression associated with high protein expression rather than the exact degree of linear correlation. We therefore defined high expression of protein or RNA as being higher than 90% of housekeeping-gene protein or RNA expression, respectively, in each sample. In clinical cancer samples from the Clinical Proteomic Tumor Analysis Consortium (CPTAC), the median accuracy of high RNA predicting high protein expression (and therefore low RNA predicting low protein expression) across cell-surface targets was 86% (IQR 79%–94%). Similarly, in cell lines from the Sanger Cell Model Passports (SCMP) (2), the median accuracy of high RNA predicting high protein expression across cell-surface targets was 97% (IQR 90%–99%). Finally, in benign tissue samples from the Human Protein Atlas (HPA) (3), the median accuracy of high RNA predicting high protein expression across cell-surface targets was 97% (IQR 90%–100%). These data demonstrate that high RNA expression can be used to accurately infer high protein expression in many tumors (Supplemental Figure 4). The inaccuracy rate (low RNA expression predicting high protein expression, or high RNA expression predicting low protein expression) can be inferred from 100% minus these rates.

### Cell-surface target expression across cancers.

We first evaluated expression of the cell-surface targets across the benign and tumor tissues in our integrated data sets. Broadly speaking, hierarchical clustering of tumor types by cell-surface target expression clustered by histology of adenocarcinoma versus non-adenocarcinoma (Figure 1B). About half of the cell-surface targets were more ubiquitous and highly expressed, including some across all tumor types such as *CD276* (B7-H3), *FN1*, and *GPNMB*. Across adenocarcinoma histology subtypes, *ERBB2* (HER2), *ERBB3* (HER3), *EPCAM*, *TACSTD2* (TROP2), and *NECTIN4* were all found to be highly expressed. Other cell-surface targets had more restricted expression by tumor type, including *FOLR1*, which was detected in gynecologic malignancies, *FOLH1* (PSMA) in prostate cancer, *PMEL* (gp100) and *TYRP1* in cutaneous and uveal melanoma, and *SCL44A4*, *CEACAM5*, and *CEACAM6* in gastrointestinal (GI) malignancies (Supplemental Figure 5). These results were confirmed in our microarray data as well (Supplemental Figure 6). Notably, these findings are concordant with the tumor type/cell-surface target pairings for which therapies directed against these targets are currently in clinical trials. In the set of benign tissues, as expected, some targets were ubiquitously highly expressed, while others showed more restricted or universally low expression (Supplemental Figure 7). Interestingly, FDA-approved agents exist for targets in each of these categories, including *ERBB2* (ubiquitous expression), *TACSTD2*, *FOLR1*, *NECTIN4* (restricted expression), and *FOLH1* (low expression).

**Figure 2:**
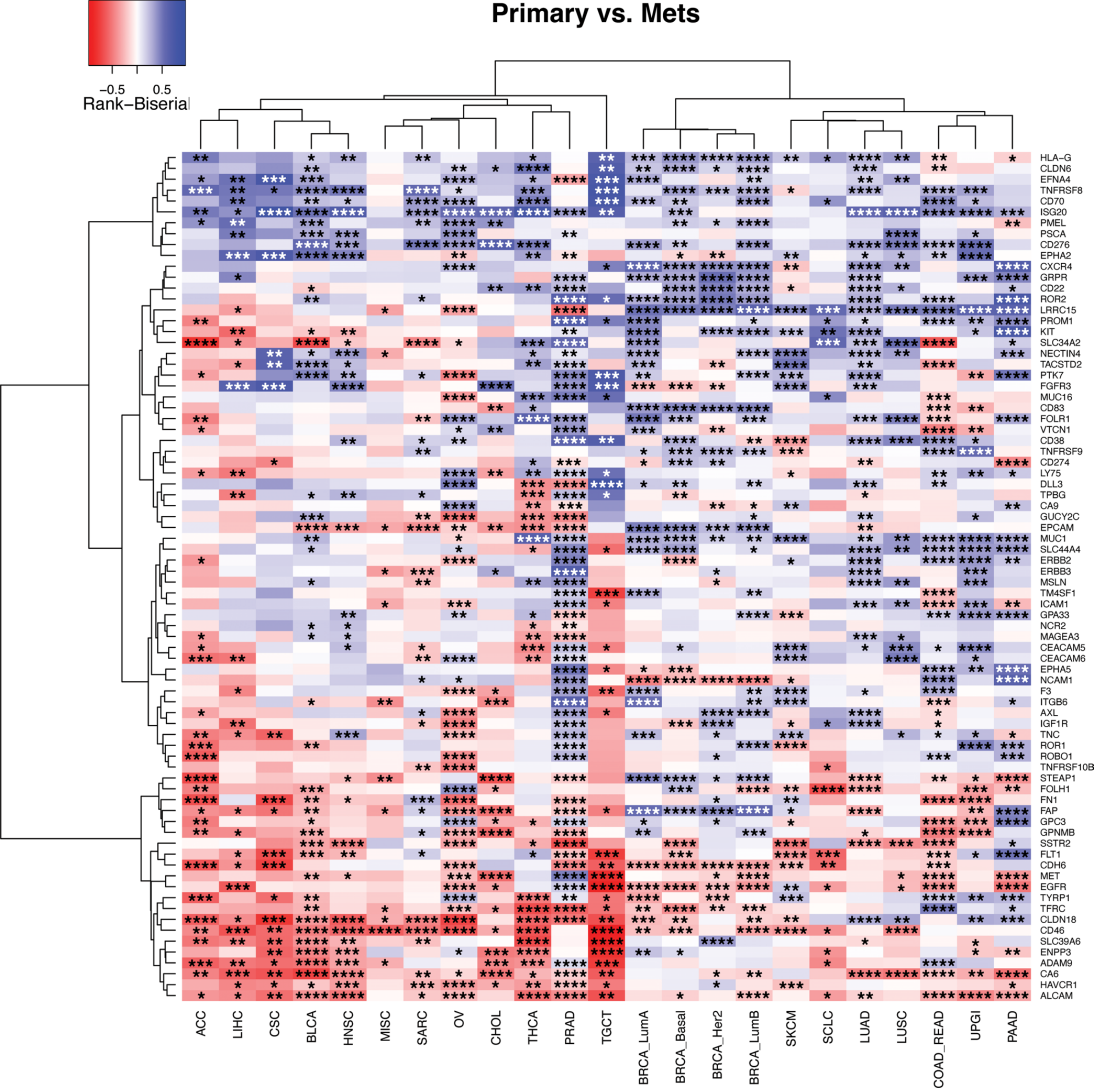
Primary versus metastatic tumor expression. A Rank-biserial correlation was calculated between the primary and metastatic samples in 23 tumor types (columns), with at least 10 samples in each group for each of the 78 cell-surface targets (rows). Within each histology, negative values (red) indicate enrichment of the target in metastatic tumors of that histology, while positive values (blue) indicate enrichment of the target in primary tumors. Hierarchical clustering was performed. Asterisks indicate Wilcoxon’s rank-sum FDR comparing primary versus metastatic expression: *P ≤ 0.05, **P ≤ 0.01, ***P ≤ 0.001, ****P ≤ 0.0001.

Currently available tumor gene expression data sets that identify cell-surface targets are predominantly derived from primary tumor samples, whereas the applications of cell-surface-directed therapies are primarily approved or investigated in the metastatic setting. This mismatch of primary tumor data with metastatic disease application represents a gap in knowledge. As such, we leveraged our metastasis-enriched gene expression data set to compare cell-surface target expression between primary and metastatic tumors within each tumor type. This analysis demonstrates that many targets had divergent expression between primary and metastatic tumors (Figure 2), which has potential clinical implications regarding therapy selection. For example, *NECTIN4*, the target of the ADC enfortumab vedotin that is FDA-approved for advanced bladder cancer and under investigation across multiple additional malignancies, has even higher expression in primary compared with metastatic tumors across most tumor types, including bladder cancer (BLCA; Figure 2). Conversely, *EGFR*, the target of a promising BiTE in early-stage trials in pancreatic cancer (4), has higher expression in metastatic compared with primary tumors across many tumor types, including pancreatic cancer (PAAD; Figure 2).

However, many targets had more mixed patterns. *ERBB2* is highly expressed in metastatic upper GI carcinomas (UPGI), for which the ADC fam-trastuzumab deruxtecan is FDA approved, but we find that *ERBB2* expression is even higher in primary tumors compared with UPGI metastases (Supplemental Figure 8). Similarly, we find that *FOLR1* has higher expression in primary compared with metastatic ovarian cancers (OV), where the FOLR1-targeting ADC mirvetuximab soravtansine-gynx is FDA approved (Supplemental Figure 8). These results could be used to guide which cell-surface-targeted therapies could be moved into earlier disease stages in situations where a clinical rationale exists. Importantly, we also identified target-histology pairs where expression was relatively low in primary tumors, but relatively high in metastatic lesions, including *MET* in cholangiocarcinoma (CHOL) and *GPNMB* in colorectal (COAD_READ) cancers (Supplemental Figure 8). All the ADCs for these targets are currently in phase II/III trials in other malignancies, and our results suggest the potential for repositioning these existing ADCs into new clinical indications. Taken together, this highlights the complexity of implementing precision medicine approaches utilizing cell-surface-targeted therapies, including the importance of evaluating target expression in the specific setting (primary versus metastatic) of proposed use, as well as the potential for drug repositioning across malignancies based on target expression.

### Potential drug-repositioning candidates.

**Figure 3:**
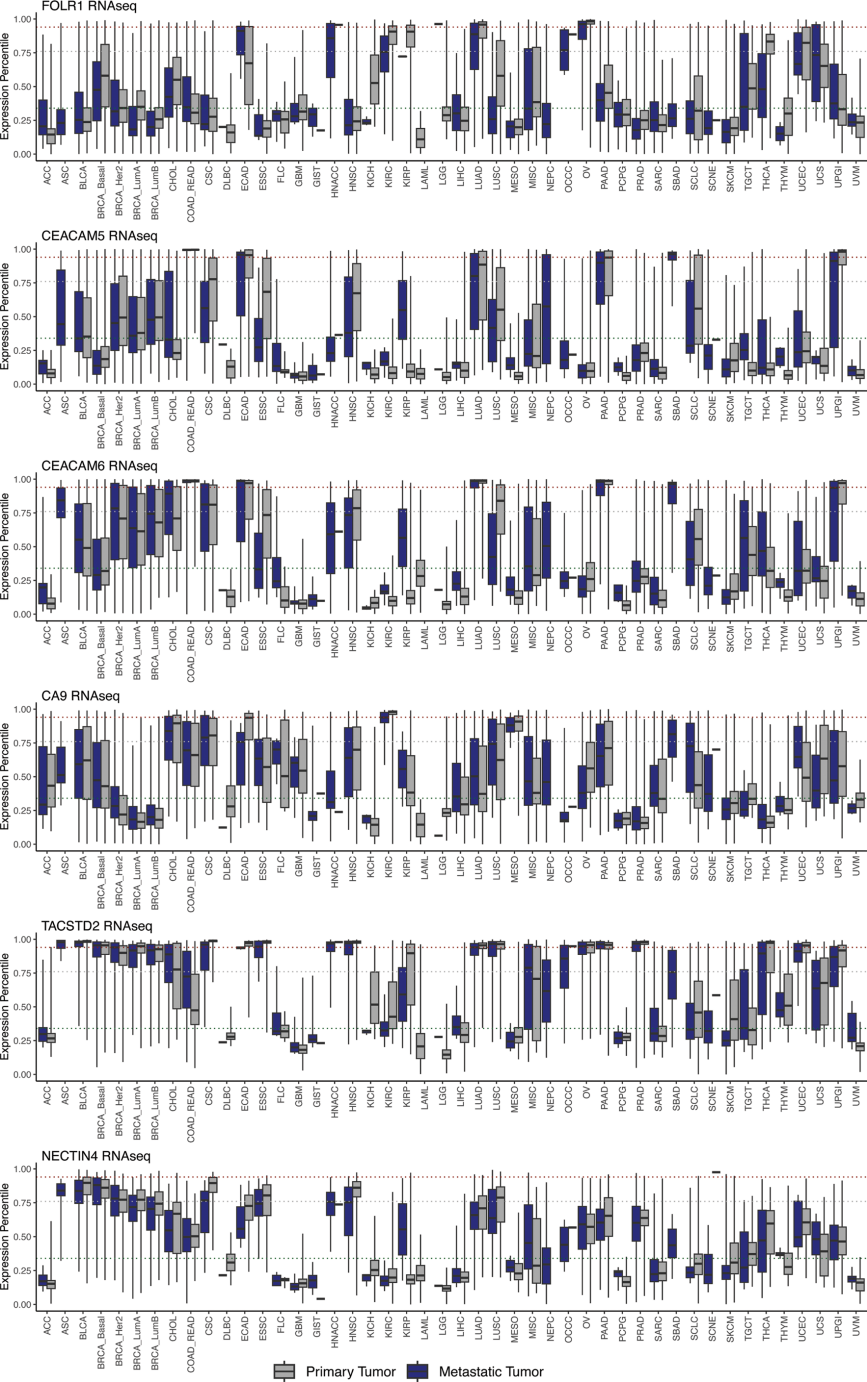
Drug-repositioning candidates. Box-and-whisker plots show the metastatic (blue) and primary (gray) cancer expression in the RNA-seq data for various cell-surface targets. RNA expression levels are percentile rank normalized, ranging from 0 to 1. Green/gray/red dotted lines: 1st/50th/90th percentiles of housekeeping gene expression. Middle line: 50th percentile (median). Lower hinge: 25th percentile. Upper hinge: 75th percentile. Whiskers: minimum and maximum.

Among the cell-surface targets with more tumor-type-restricted expression, our pan-cancer approach identifies associations beyond FDA-approved tumor indications, which suggests new avenues for repositioning existing FDA-approved or previously investigated cell-surface-targeted therapies. As expected, we find that *FOLR1* is highly expressed in ovarian cancer (OV), where a FOLR1 ADC is FDA approved, and in NSCLC (LUAD), where FOLR1 ADCs are under investigation (Supplemental Table 1). However, we also note high *FOLR1* expression in endocervical adenocarcinoma (ECAD), and in clear cell renal cell carcinoma (KIRC) (Figure 3 and Supplemental Figure 9). *CEACAM5/6* expression is elevated in GI malignancies where CEACAM5/6 ADCs are under investigation (COAD_READ, UPGI, SBAD, PAAD), but we also observe high expression in both NSCLC (LUAD) and head and neck squamous cell cancers (HNSC) (Figure 3 and Supplemental Figure 9). Finally, the CA9 RPT ^177^Lu-DPI-4452 is under investigation for RCC (KIRC), pancreatic cancer (PAAD), and colorectal cancer (COAD_READ), all of which are supported by our expression data (Figure 3 and Supplemental Figure 9). However, other tumor types that may have high expression of *CA9* include cholangiocarcinoma (CHOL), cervical squamous cell cancer (CSC), mesothelioma (MESO), and even small bowel adenocarcinoma (SBAD). Our approach also suggests candidate histologies for the repositioning of therapies targeting more ubiquitous cell-surface targets. For instance, we find that *TACSTD2* (TROP2) is broadly expressed across adenocarcinomas beyond those where TROP2 ADCs are FDA approved or under investigation, including gynecologic (ECAD, OV, UCEC) and GI malignancies (PAAD, UPGI) (Figure 3 and Supplemental Figure 9). Similarly, we observe that *NECTIN4* is not only expressed highly in bladder cancer where it has an FDA-approved ADC, but also across breast cancer subtypes (BRCA), NSCLC (LUAD, LUSC), and some head and neck (HNSC) and cervical (CSC) squamous cell cancers (Figure 3 and Supplemental Figure 9). These data also reveal that for most cancer types, even when average expression for a cell-surface target is low, there are usually at least some tumors with extremely high outlier expression, supporting the rationale for tumor agnostic/basket clinical trials that enroll target-positive tumors regardless of the underlying histology.

### Identification of potential cell-surface targets from clinical RNA-seq.

As more cell-surface therapies advance in clinical trials and enter clinical practice, a high-throughput approach to infer target protein expression positivity across cancer types can help guide treatment and clinical trial selection. For instance, patients with advanced metastatic HER2-low breast cancer are eligible for both the TROP2-targeted ADC sacituzumab govitecan and the HER2-targeted ADC fam-trastuzumab deruxtecan (5). Clinicians currently do not have biomarkers to guide the selection of one over the other for an individual patient, which is particularly crucial given emerging data showing poor clinical outcomes with sequential use of these 2 ADCs (6). As more cell-surface-targeted trials and therapies become available, this problem will only be exacerbated. Clinical whole-transcriptome RNA-seq is entering clinical practice, with multiple commercial options now available, and represents a potential approach to screen expression of cell-surface targets at an individual patient level to address this unmet need. We have therefore created a website (https://www.humonc.wisc.edu/cell-surface-target-expression/; password: zona-gale) that allows a percentile score for any cell-surface target (calculated for a particular patient’s tumor using only their RNA-seq data as described in the Methods) to be compared against pan-cancer distributions in order to identify potential highly expressed cell-surface targets. We show above that high gene expression accurately predicts high protein expression for most cell-surface targets, suggesting that these data could be used for screening potential targets to be confirmed with immunohistochemistry (IHC), a requirement for many trials.

### Cell-surface target cell line model system matching.

**Figure 4:**
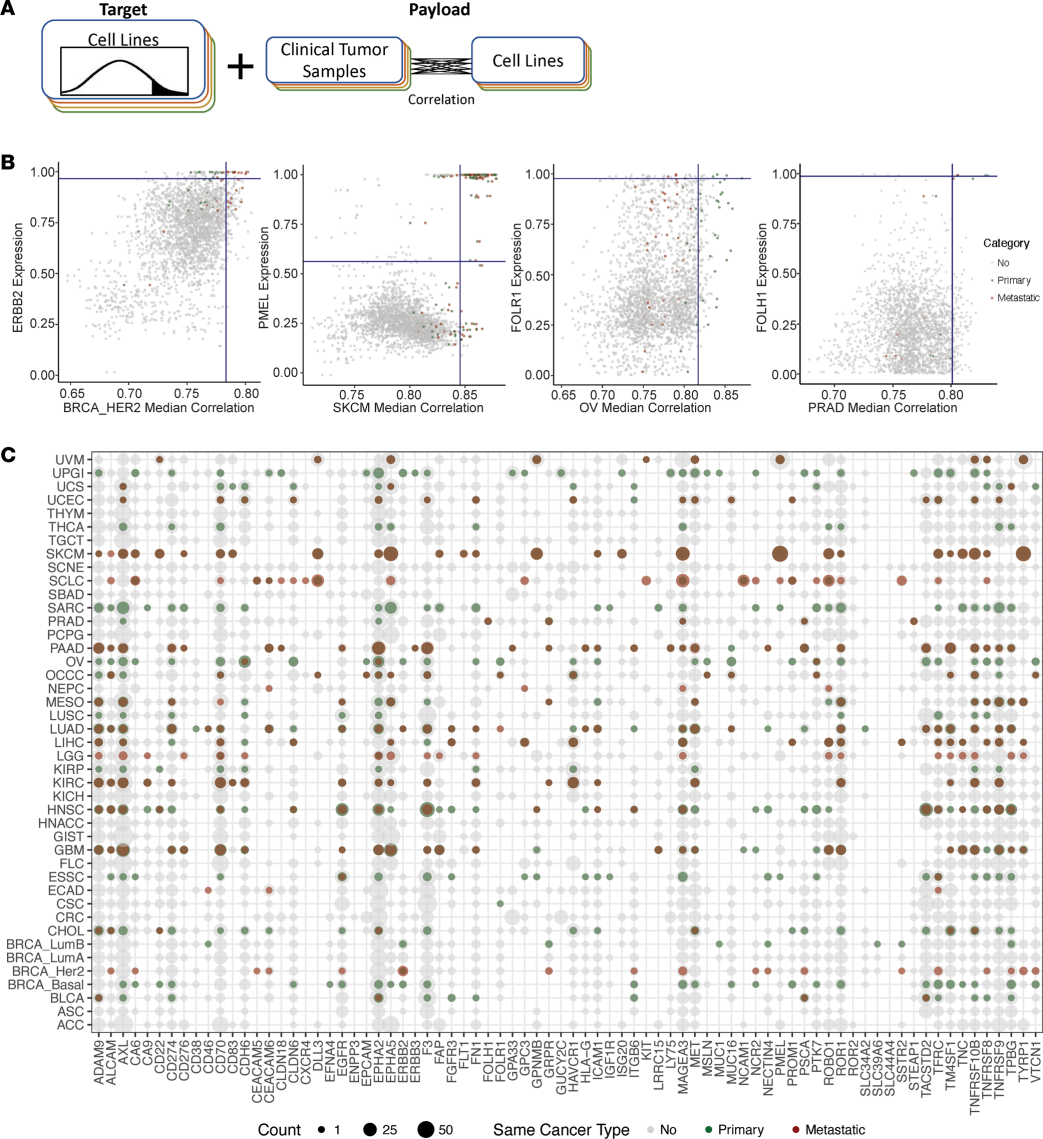
Selecting a cell line model system. (**A**) Schematic of rationale for identifying optimal cell line based on both cell-surface target expression and correlation with high-purity clinical tumor samples of the intended patient population. (**B**) Number of cell lines that met the following criteria: (i) cell lines with expression that was in the 95th percentile of expression or greater for each target in the tumor RNA-seq (horizontal blue line) and (ii) 95th percentile or greater of correlation with tumor samples of each primary/metastatic tumor type (vertical blue line). Gene expression for each cell line was correlated with each high-purity primary/metastatic cancer sample, and a median was calculated for each cancer type. Each data point represents 1 cell line target expression (*y* axis) versus the median correlation of that cell line to a primary or metastatic tumor type (*x* axis). Red/green point = cell lines of the same cancer type as the metastatic/primary cancer tissue sample that meet criteria i and ii. Gray dot = cell lines of a different cancer type that meet criteria i and ii. (**C**) Number of cell lines (indicated by dot size) that meet criteria i and ii across tumor types (rows) and targets (columns). Green and red dots represent cell lines that are the same cancer type for primary and metastatic cancer, respectively. Gray dots represent cell lines of a different cancer type.

To assess the potential for drug repositioning, appropriate cell line models for in vitro and subsequent in vivo investigations are needed. However, these can be challenging to identify, since both expression of the cell-surface target and sensitivity to the payload should ideally match that of the clinical tumor population. To address this challenge, we have created a framework for matching the best cell line for each target and tumor type, incorporating both these parameters (Figure 4A). First, we identified cell lines with expression that was in the 95th percentile of expression or higher for each target in the tumor RNA-seq. Next, we correlated every high-purity tumor sample RNA-seq with every cell line, and identified cell lines that were in the 95th percentile of correlation or higher with each tumor type. To validate our approach, we evaluated several specific examples (Figure 4B). For *ERBB2* (HER2) in the HER2 breast cancer samples (BRCA_HER2), most of the top matching cell lines identified were HER2 breast cancer cell lines matched with metastatic HER2 breast cancer samples. For *PMEL* in the cutaneous melanoma (SKCM) samples, most of the top matching cell lines identified were melanoma cancer cell lines matched with melanoma tumor samples. For *FOLH1* (PSMA) in the prostate adenocarcinoma cancer samples, most of the top matching cell lines identified were prostate cancer cell lines matched with prostate cancer tumor samples. Overall, the majority of cell-surface targets had at least one cell line matching these criteria (Figure 4C), which we have provided as a reference and resource (Supplemental Table 4). In rare tumor types with few/no cell line models, this could be used to find an approximation for in vitro studies. Alternatively, in tumor types with many cell lines available, this could be used to prioritize.

### Single-cell heterogeneity of cell-surface targets in tumor tissue.

**Figure 5:**
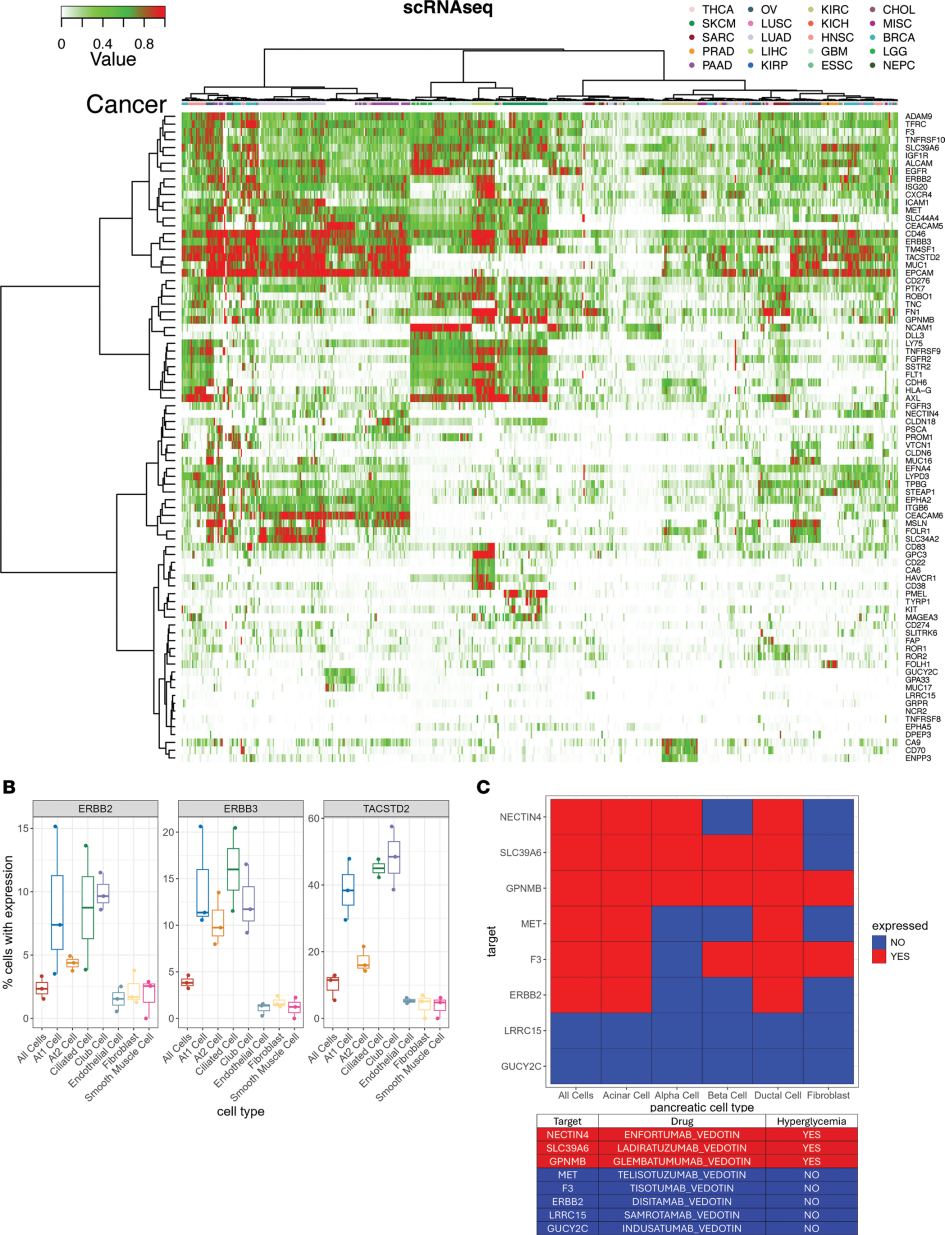
Single-cell heterogeneity. (**A**) Heatmap of the proportion of the tumor cells in each scRNA-seq sample that expresses each cell-surface target. Rows are targets and columns are scRNA-seq samples. Red represents samples with a high proportion of tumor cells expressing each target, green represents samples where a more mixed proportion of tumor cells express each target, and white represents an absence of cell-surface target expression. (**B**) Percentage of cells positive for ERBB2, ERBB3, and TACSTD2 across all cell types and within each cell type subpopulation in replicate lung scRNA-seq samples. Middle line: 50th percentile (median). Lower hinge: 25th percentile. Upper hinge: 75th percentile. Whiskers: minimum and maximum. (**C**) Detection of vedotin-conjugate target expression across different pancreatic cell types showing that target expression in α cells correlates with presence or absence of hyperglycemia as a clinical toxicity.

Heterogeneity in tumors is the rule, not the exception, and is a major driver of treatment resistance in metastatic disease. This is an important consideration with respect to payload selection and mechanism of action. Cell-surface-targeted ADCs and RPTs are designed to deliver a payload to a region surrounding the targeted cells, and thus their efficacy may be less impacted by the heterogeneity of tumors. Conversely, the efficacy of immunomodulatory cell-surface-targeted therapies such as CARs and BiTEs may be adversely impacted by heterogeneity of target expression. We utilized scRNA-seq data to assess the degree of variability in cell-surface target expression across 558 primary and metastatic tumor samples across a wide range of histologies, focused on the tumor cell component of each sample. Interestingly, while some cell-surface targets had relatively uniform expression on most tumor cells in each sample, the majority of cell-surface targets demonstrated a large degree of heterogeneity (Figure 5A). We next examined several specific FDA-approved indications for cell-surface therapies that are not guided by IHC, to evaluate the potential impact of tumor heterogeneity. *TACSTD2* (TROP2) was fairly uniformly expressed in basal breast cancer samples, detected in a median of 94% of tumor cells. *PMEL* was likewise detected in a median of 93% of tumor cells, though some samples exhibited low rates of positivity. In contrast, *FOLH1* (PSMA) expression was much more heterogeneous, expressed in a median of 44% of tumor cells in prostate cancer samples, and *FOLR1* was expressed in a median of 53% of tumor cells in ovarian cancer samples (Supplemental Figure 10). Clinically relevant, this heterogeneity may impact not only treatment response and the development of acquired resistance through selection for cells without target expression, but the consideration of what type of payload might be most efficacious for a particular cell-surface target.

### Single-cell heterogeneity of cell-surface targets in benign tissue and toxicity.

While intratumoral heterogeneity of target expression could impact tumor treatment responses, differences in target expression in various cellular subpopulations could impact toxicity. Most FDA-approved ADCs have toxicities such as cytopenias, nausea/vomiting, and peripheral neuropathy typically seen with standard cytotoxic chemotherapies and are presumed to be due to systemic effects of the cytotoxic chemotherapy payload. However, many of these agents also have toxicities that are not typical of standard cytotoxic agents and could be related to delivery of the payload to normal tissues expressing the cell-surface target. For instance, *ERBB2* (HER2)–targeted ADC fam-trastuzumab-deruxtecan, FDA approved for HER2-expressing breast cancer and NSCLC, has a well-described risk of interstitial lung disease (ILD), which can be seen in up to 10% of patients, and can be fatal (7). This is also seen with *ERBB3* (HER3)– (8, 9) and *TACSTD2* (TROP2)–targeted (10) deruxtecan conjugates. However, the mechanistic contribution of payload type versus cell-surface target expression remains poorly understood. We evaluated *ERBB2*, *ERBB3*, and *TACSTD2* expression in scRNA-seq of the normal lung in our data set and identified enrichment of cells expressing all 3 targets among AT1, ciliated, and club cell populations, in contrast with other cell types (Figure 5B). As this pulmonary toxicity is not seen with govitecan conjugates targeting TROP2 (sacituzumab govitecan) (11, 12) or HER2 (disitamab vedotin) (13), the ILD phenotype is likely driven by the specific combination of the deruxtecan payload and target expression on these lung cell populations. We also leveraged our data set to investigate a unique toxicity of enfortumab vedotin, an FDA-approved NECTIN4-targeting ADC, which carries a risk of severe hyperglycemia, seen in up to 8% of patients (14). This is also seen with ladiratuzumab vedotin targeting SLC39A6 (15) and glembatumumab vedotin targeting GPNMB (16). However, it is not seen with any other solid tumor vedotin-conjugate ADCs with published toxicity data, including telisotuzumab vedotin (MET) (17), tisotumab vedotin (tissue factor) (18), disitamab vedotin (HER2) (13), indusatumab vedotin (GUCY2C) (19) or samrotamab vedotin (LRRC15) (20). When we investigated expression of these targets in scRNA-seq of the normal pancreas, we found that target detection on pancreatic α cells, but not other pancreatic cell types, correlated with the presence or absence of hyperglycemia as a toxicity across these vedotin-conjugate therapies (Figure 5C). As pancreatic α cells play a key role in glucagon secretion, this suggests that on-target binding and/or payload release of the respective ADCs to these cells may lead to glucagon release to drive the acute hyperglycemia seen with these agents.

### Combinations of cell-surface targets can improve cancer specificity.

**Figure 6:**
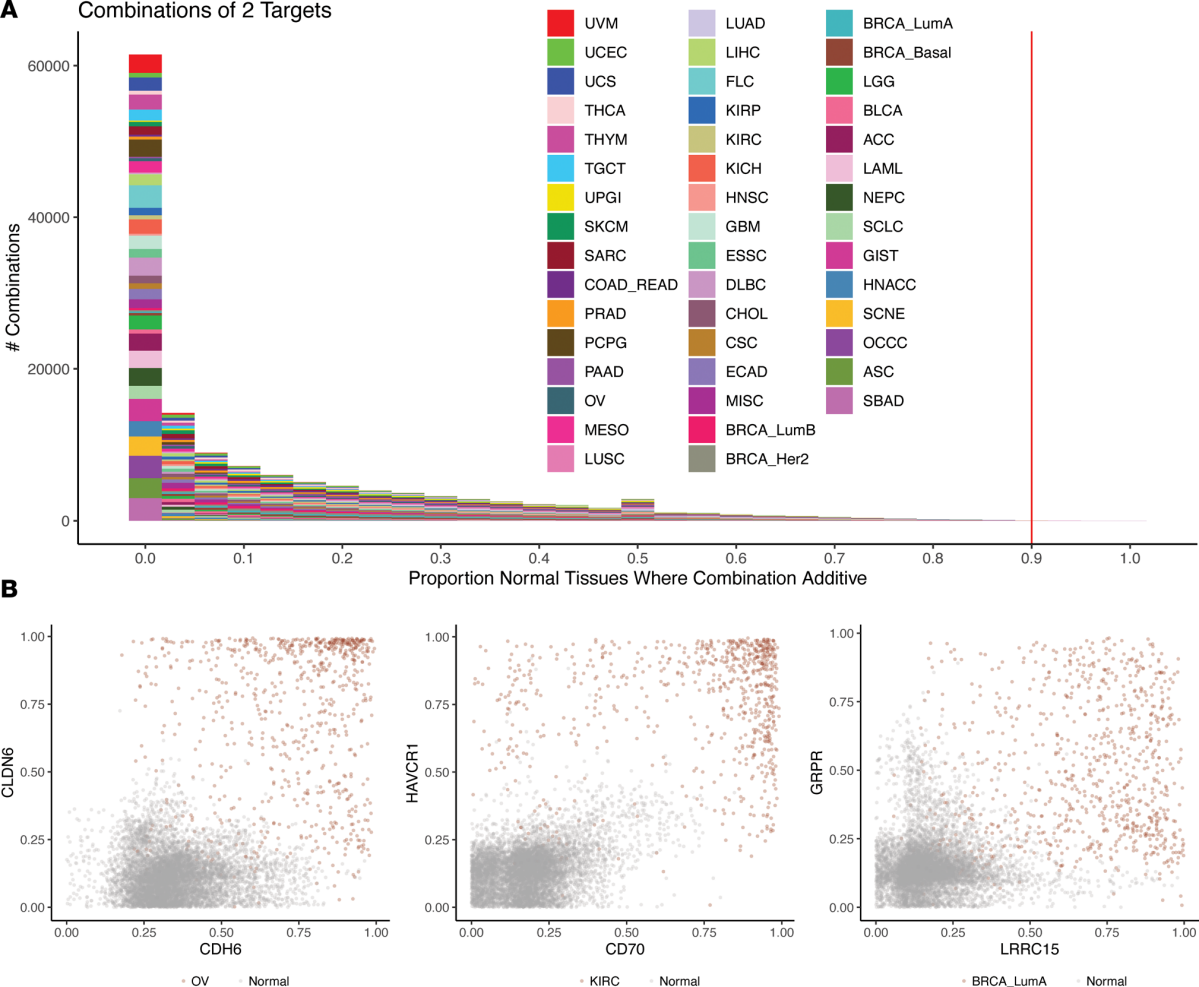
Combinations of 2 targets. (**A**) We created logistic regression models for every pair of cell-surface targets, comparing each cancer type and each normal tissue type. We then only retained pairs where (i) expression was higher in cancer versus normal and the combination had good discriminative power, with an F1 score of greater than 0.95; and (ii) each individual gene was contributing independently, with both having Wald test *P* values (corrected for multiple testing) of less than 0.05. The number of combinations as a function of the proportion of cancer-normal tissue comparisons meeting these 2 criteria is shown. The red line indicates cell surface target combinations where greater than 90% of the cancer-normal tissue comparisons met criteria i and ii, and is where the examples shown in **B** are drawn. (**B**) Example combinations of how gene expression percentiles of 2 cell-surface targets (*x* and *y* axes) can better stratify benign samples (gray) from cancer samples (red).

Historically, cell-surface-targeted therapies have relied on monovalent antibodies. However, combinatorial approaches can potentially improve cancer specificity and decrease normal tissue exposure. Emerging approaches allow for bivalent combinations of cell-surface targets. Several bispecific ADCs and CAR-Ts are under investigation in clinical trials (Supplemental Table 1). However, identifying appropriate combinations empirically presents challenges. We therefore sought to evaluate all combinations of 2 clinical cell-surface targets in their combinatorial differential expression between cancers and normal tissues. To do this, we created logistic regression models for every pair of cell-surface targets, comparing each cancer type and each normal tissue type. We then only retained pairs where (a) expression was higher in cancer versus normal and the combination had good discriminative power with an F1 score of greater than 0.95, and (b) each individual gene was contributing independently, with both having Wald test *P* values (corrected for multiple testing) of less than 0.05. Overall, only a small minority of cancer-normal tissue pairs across cancer types met these stringent criteria (Figure 6A), but these represent potential promising combinatorial cell-surface strategies. Several examples of bivalent combinations of clinical cell-surface targets demonstrate a markedly improved ability to stratify cancers from normal tissues (Figure 6B), suggesting that this approach may improve the therapeutic window and selectivity of agents directed against more ubiquitously expressed targets.

## Discussion

While most cell-surface-targeted therapies are undergoing initial development in metastatic disease, prior studies evaluating cell-surface target expression across tumor types have predominantly focused on primary tumor target expression, due to the more limited availability of metastatic tumor biopsies. We have directly addressed this key gap through the curation of a large pan-cancer and normal tissue gene expression data set including over 6,000 metastatic tumors. To our knowledge, this represents the largest integrated data set of metastatic tumor tissue gene expression focused on cell-surface target expression reported to date. Leveraging this unique resource, we are able to provide a comprehensive overview of cell-surface target gene expression in primary and metastatic solid tumors with a focus on cell-surface targets with existing FDA-approved or investigational cell-surface-targeted therapies. Our analysis demonstrated a number of targets with differential expression between primary and metastatic disease, with the potential to guide avenues of future clinical development for therapies directed against these targets. Additionally, we identified multiple opportunities for repositioning existing FDA-approved and investigational cell-surface-targeted therapies to new tumor types, as well as an approach to identifying appropriate in vitro models for drug repositioning.

Leveraging tumor scRNA-seq data, we found that intratumoral heterogeneity varied across surveyed targets, including those with FDA-approved indications. ADCs and RPTs likely provide some level of bystander effect that can potentially overcome heterogeneity. However, higher heterogeneity of target expression could still lead to selection for non–target-expressing tumor cells as a mechanism of acquired resistance to these therapy classes. CAR-Ts and BiTEs, which drive immune responses to the target antigen, may be more sensitive to target expression variability within a tumor. It is notable that to date these agents have been most successful in hematologic malignancies subject to ubiquitous and obligate expression of the cell-surface target. These principles also apply to normal tissues, where selective enrichment of a target in a critical cell population could lead to clinically relevant toxicities from particular target/payload combinations, as we have identified in both normal lung and pancreas through an analysis of normal tissue scRNA-seq. Taken together, our findings emphasize the importance of incorporating scRNA-seq in the identification of potential cell-surface targets, as well as in the understanding of toxicities that emerge.

Our study is not without limitations. Due to well-established potential for dropout in scRNA-seq, the scRNA-seq data were binarized (21–23); while the correlation between these binarized values and protein expression is not well understood, these results do illustrate the influence of heterogeneity of target gene expression at least at the transcriptional level. The aggregation of bulk RNA-seq data across multiple studies comprising samples of varying quality and techniques is not equivalent to a single uniform data set. Given the overarching goals of this study, we prioritized broader inclusion of available samples to improve our sampling of rare cancer types. Nonetheless, the high expression of cell-surface targets in the tumor types, which we would expect, are reassuring. Additionally, we were able to validate in a set of matched samples that high gene expression using this approach accurately predicts high protein expression for our panel of cell-surface targets. However, the degree of protein expression required for effective cell-surface-targeted therapy is unknown and the efficacy of cell-surface-targeted therapy ultimately depends not only on target engagement, but also linker-dependent payload delivery and tumor payload sensitivity. For instance, in the phase I/II pan-cancer trial of sacituzumab govitecan, where hormone receptor–positive and triple-negative breast cancer (BRCA) demonstrated clinical benefit rates of 44.4% and 45.4%, respectively, other tumor types identified in our analysis with high *TROP2* expression had more variable clinical benefit rates, ranging from 0% for pancreatic adenocarcinoma (PAAD) to 21.1% for esophageal carcinoma (UPGI) to 44.4% for endometrial cancer (UCEC) (24). Similarly, recently reported phase II studies of the *NECTIN4* ADC enfortumab vedotin have demonstrated antitumor activity in tumor types identified in our analysis as having high *NECTIN4* expression; however, response rates were variable and in all cases lower than the 40%–50% overall response rate seen in urothelial cancer (BLCA) (25). In triple-negative and hormone receptor–positive breast cancer, antitumor activity was demonstrated with overall response rates of 19% and 15.6%, but did not meet prespecified thresholds to continue with further development (26). In NSCLC, antitumor activity was seen in the adenocarcinoma (LUAD) cohort, but not in the squamous (LUSC) cohort (27). Taken together, this highlights the complexity of predicting tumor response and resistance to effective cell-surface-targeted therapies, though the presence or absence of target expression will remain an important component of this process.

With the advent of bispecific antibodies and beyond, combinatorial approaches are beginning to gain traction, and offer a potential way to improve the therapeutic ratio of cell-surface-targeted therapies, increasing tumor specificity and reducing toxicities related to on-target gene expression in normal tissues. However, selection of appropriate target combinations empirically is exceedingly challenging, and we describe a computational methodology to identify potential combinations with the highest likelihood of efficacy while minimizing on-target toxicities. As more advanced cell-surface-targeting technologies move forward, particularly those leveraging immunomodulatory payloads, we hope that this resource could be used to identify unappreciated combinations, such as the ones we illustrate.

Finally, as clinical indications for cell-surface-targeted therapies expand, precision oncology biomarkers for selection of the most appropriate therapy for an individual patient are needed. RNA-seq, in addition to DNA sequencing, is becoming more mainstream in clinical practice, with multiple commercial options. This poses a challenge for oncologists to interpret, as clinical indications have not yet begun to incorporate RNA expression of specific targets. While RNA and protein levels are moderately correlated, it is still not a perfect surrogate. However, RNA-seq could be used to screen for potential highly expressed surface targets, which could then be confirmed by IHC. A high-throughput method of identifying potential cell-surface targets will become increasingly important as more of these therapies enter clinical trials and practice, as it will not be practical to stain for all possible targets. Herein, we have also provided a reference of the distributions of every cell-surface target, which could be used as a resource by oncologists or molecular tumor boards. Since the percentile for any target can be calculated using only an individual patient’s RNA-seq profile, these data could be used to help guide the subset of targets to confirm with IHC.

## Methods

### Sex as a biological variable.

Our study evaluated tumor samples from both male and female patients in publicly available gene expression data sets.

### Clinical landscape of cell-surface protein targets in adult solid tumors.

We utilized ClinicalTrials.gov to compile a comprehensive list of interventional clinical trials (as of October 31, 2023; Supplemental Table 1) on various targeted cancer therapies for adult solid tumors. The targeted therapies include ADCs, BiTEs, CAR-T, and other CAR variants such as CAR-NK, CAR-DC, CAR-M, and RPT. We manually curated the cell-surface protein targets in each trial based on information from trial details and related publications. For phase annotation, we only considered the most advanced phase in the case of multiphase trials. For instance, trials in phases I/II were categorized as phase II, while those in phases II/III were considered phase III trials. Targets were excluded from subsequent analyses if there was not a specific gene coding for the protein, the absence of these genes in all data sets, or as part of a NOT-gate CAR-T design. In addition, targets that had only a single ended trial without any additional trials were excluded.

### Bulk tumor RNA-seq and microarray data.

We have compiled gene expression data from a large collection of RNA-seq (*N* = 52) and microarray (*N* = 85) studies. These studies encompass various cancer types, normal tissues, and cell lines. The data sets were downloaded from multiple sources, including the NCBI Gene Expression Omnibus (GEO), cBioPortal, and supplementary files from individual publications, among others (Supplemental Table 2). To standardize gene symbols across various versions of gene annotations used in different studies, we converted different gene identifiers (Ensembl gene ID, Entrez gene ID, RefSeq gene ID, Gene Atlas, etc.) to official HGNC gene symbols using the R packages biomaRt and AnnotationDBi (https://www.r-project.org/). Only protein-coding genes with valid HUGO gene symbols were retained. For microarray data, the corresponding probe annotation file for a given platform was downloaded using the R package annotate. In cases where multiple probes mapped to the same gene, we selected the probe with the highest average expression across samples.

Clinical data were primarily downloaded using the R package GEOquery, unless otherwise provided. We extracted relevant clinical information, including cancer type and primary or metastatic tumor. Cancer types were annotated in accordance with TCGA cancer type abbreviations (Supplemental Table 3). We performed subtyping analyses on breast cancer samples and cancer cell lines into luminal A, luminal B, HER2-rich, and basal subtypes using the R package AIMS. In cases where the “normal-like” subtype was initially assigned, we selected the second-highest-scoring subtype as the subtype assignment.

We integrated all 52 RNA-seq studies, retaining only the 17,560 protein-coding genes common to all. The resulting combined RNA-seq data set comprises these genes across 25,582 samples, including 7,927 normal tissue samples, 12,398 primary tumors, 3,807 metastatic tumors, and 1,450 cell lines. We implemented a sample-wise rank transformation (ties.method = “random”), assigning ranks from 1 to 17,560, with higher ranks indicating increased gene expression. Subsequently, we converted these ranks to percentiles by dividing each rank by the maximum of 17,560, thereby standardizing gene expression to a range from 0 to 1 for each gene in every sample.

Due to the variable number of genes across the microarray studies, we combined all 85 data sets using a union set of protein-coding genes identified in each study in order to preserve maximum information. This yielded a combined microarray data set containing 19,200 genes across 6,785 samples, including 4,468 primary tumors and 2,317 metastatic tumors. Similarly to RNA-seq data, we implemented a sample-wise rank transformation (ties.method = “random”).

### Housekeeping gene references.

We used housekeeping genes as references to assess the expression levels of target genes (21). We calculated median RNA expression distribution of housekeeping genes in each RNA-seq study included in the study. Within each RNA-seq study, the 1st, 50th, and 90th percentiles of all housekeeping gene expression were calculated. The median values of these percentiles across the studies were used as thresholds of low, medium, and high gene expression levels.

Because of variable numbers of genes on the disparate microarray platforms used, in order to make values comparable across samples from different studies, we then instead examined the percentage of housekeeping genes with lower expression than each gene. For example, a gene with expression greater than 90% of housekeeping genes in a given sample would be assigned a value of 0.9. This method is relatively insensitive to missing genes given the large number of housekeeping genes.

### scRNA-seq data.

To further investigate the heterogeneity of target expression in tumors and normal tissues, we utilized 2 scRNA-seq data sets. The first data set is the Human Cell Landscape, a pan-tissue scRNA-seq data set (22), which includes 57 normal tissue samples across 36 normal tissue types, with replicates in several normal tissues. The second data set is the Curated Cancer Cell Atlas of Tumors, a comprehensive curated pan-cancer scRNA-seq data set (23). Only data sets on adult solid tumors were used, which include 558 tumor samples across 27 cancer types and 53 studies. The primary versus metastatic origin of each sample was not available. The scRNA-seq cell-by-gene count matrices and cell annotation files were integrated by aligning cell IDs, and the scRNA-seq count matrix was converted into a binary matrix to indicate gene expression’s measurability or detectability by setting any non-zero expression value as 1 (24–26). Our analysis was exclusively focused on the cell-surface protein targets identified in the clinical trials.

### RNA and protein expression.

To investigate RNA-protein expression correlation, we utilized paired RNA and protein expression data from 3 different sources, including normal tissues from the HPA (3) (*N* = 29), primary tumors from the CPTAC (https://pdc.cancer.gov/pdc/cptac-pancancer; *N* = 1,075), and cell lines from the SCMP (2) (*N* = 942). We converted various gene identifiers to HGNC gene symbols, retaining only those protein-coding genes common to both RNA and protein expression data sets. Both RNA and protein data sets underwent percentile rank transformation. High expression of a gene was defined per sample as being greater than 90% of housekeeping genes.

### Cell line models.

We selected cell line models that are most representative for a specific cancer type based on strong correlations from transcriptomic profiling and high cell-surface protein target expression. Spearman’s correlation was calculated for each tumor sample exhibiting high tumor purity (≥0.6, as recommended by TCGA consortium) against each cell line (solid tumors with clearly defined cancer type annotations only). Tumor purity was estimated using the ESTIMATE algorithm (27) via the R package tidyestimate.

### Statistics.

Statistical tests used are indicated in each figure legend. A *P* value of 0.05 was used to determine significance. All tests were 2-sided.

### Data availability.

Data set accession numbers/sources can be found in Supplemental Table 2. scRNA-seq data were obtained from https://db.cngb.org/HCL/ and https://www.weizmann.ac.il/sites/3CA/ All data are available per these sources and repositories. Raw data are provided in the supplemental Supporting Data Values file. All other code and data sharing requests will require institutional review and a data sharing agreement.

## Author contributions

SGZ conceived of and designed the study. All authors were responsible for acquisition, analysis, or interpretation of the data. All authors contributed to drafting the manuscript and reviewing it critically for important intellectual content, and all gave final approval of the version to be published. All authors are accountable for all aspects of the work.

## Supplementary Material

Supplemental data

Supplemental tables 1-4

Supporting data values

## Version 1 09/24/2024

Electronic publication
