# Supplementary material for: Clinical cell-surface targets in metastatic and primary solid cancers
Source: JCI Insight. 2024 Sep 24;9(18):e183674. doi: 10.1172/jci.insight.183674 (PMC11457844; doi:10.1172/jci.insight.183674)
Supplement: Supplemental data [file jciinsight-9-183674-s067.pdf]

SUPPLEMENTAL FIGURES

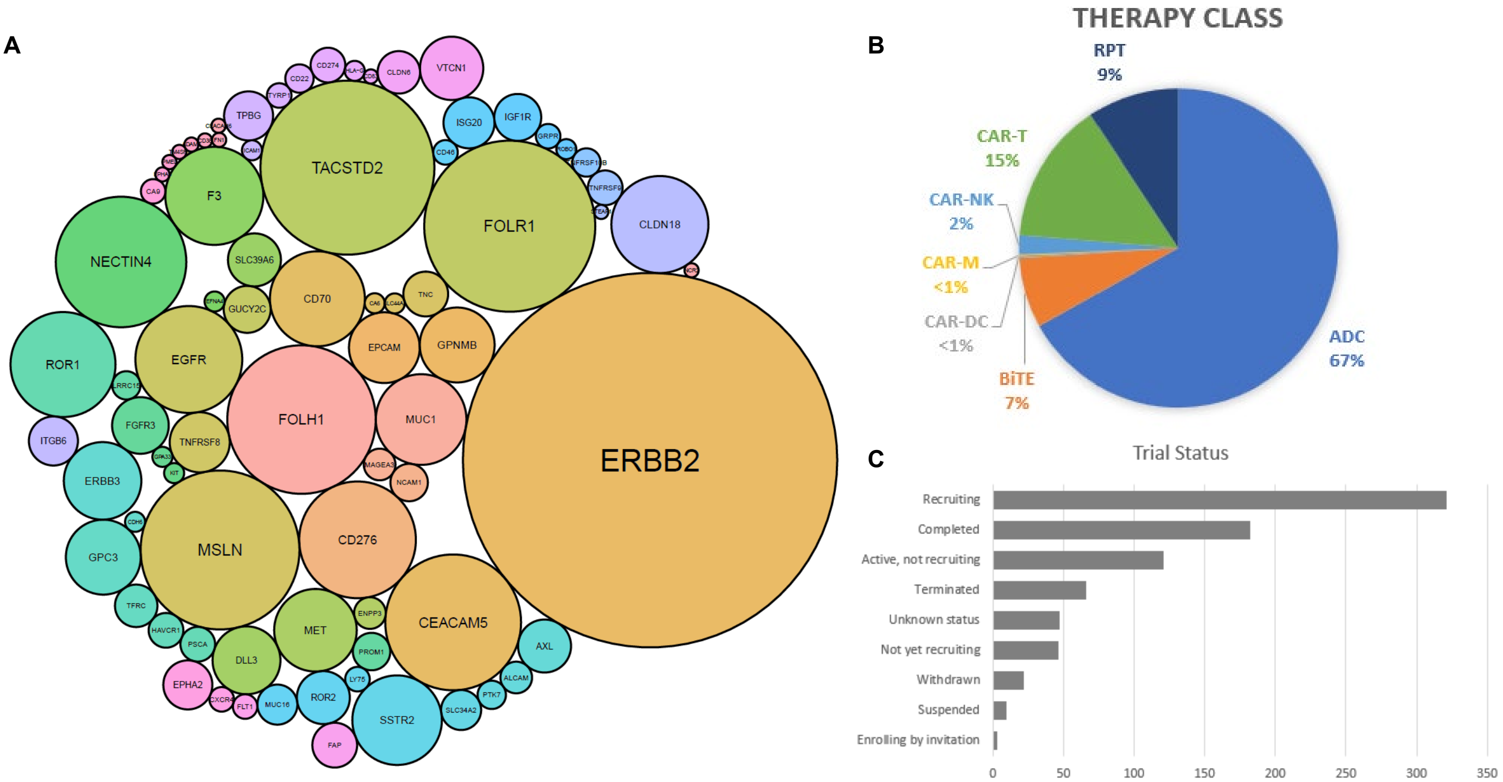

Figure S1: Clinical trials:
Landscape of cell surface protein targets in clinical trials for cell surface targeted cancer therapies. (A) The number of clinical trials for each of the 78 cell surface targets. (B) The distribution of targeted cancer therapy types across the trials. (C) The number of clinical trials by their current status (as of Oct 31st, 2023).

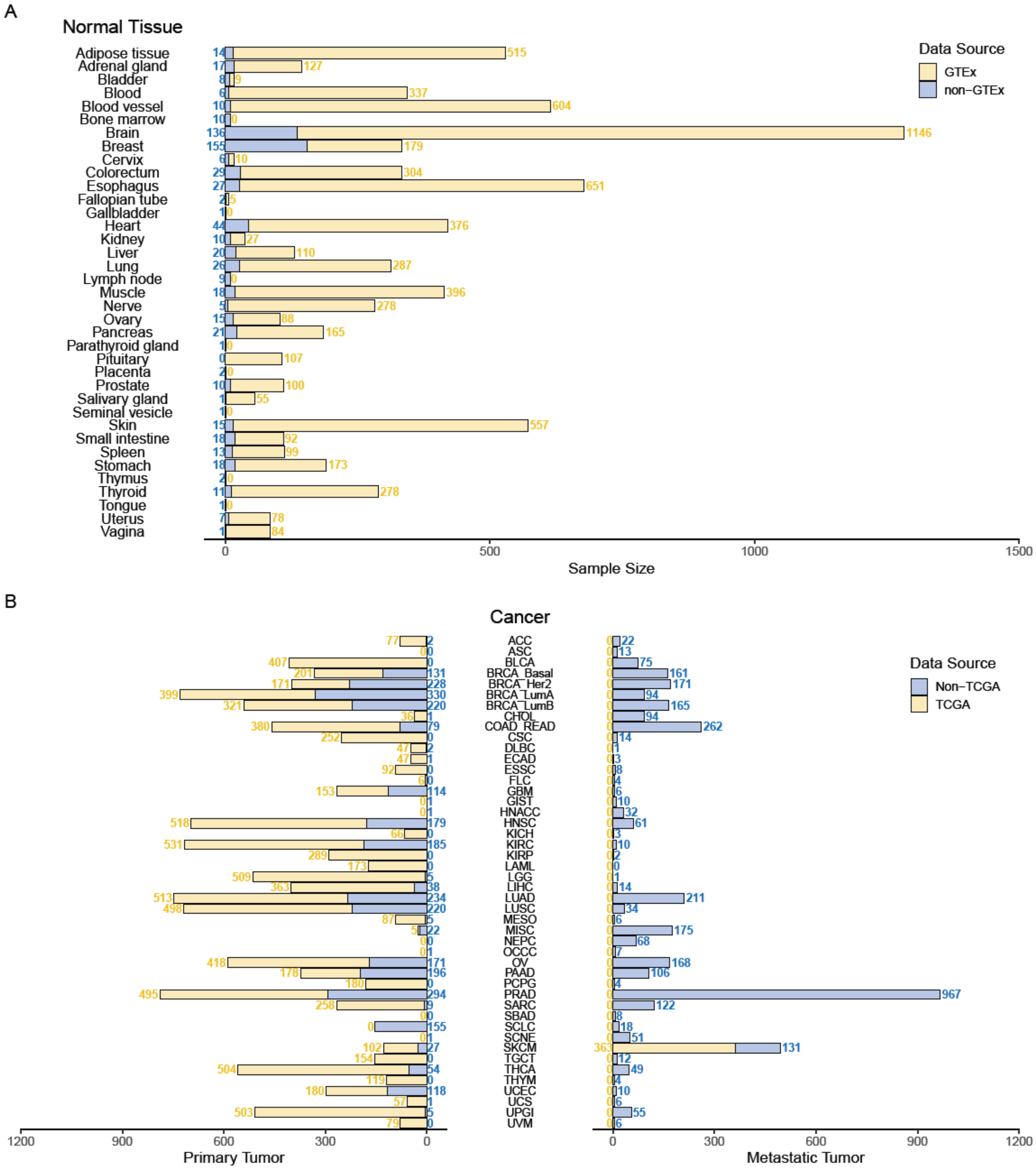

Figure S2: RNA-seq Sample Size

Sample sizes for each normal tissue type (A) and each cancer type (B), divided into primary tumors and metastatic tumors. The sources of these RNA-seq samples are specified, including GTEX, non-GTEX, TCGA, and non-TCGA.

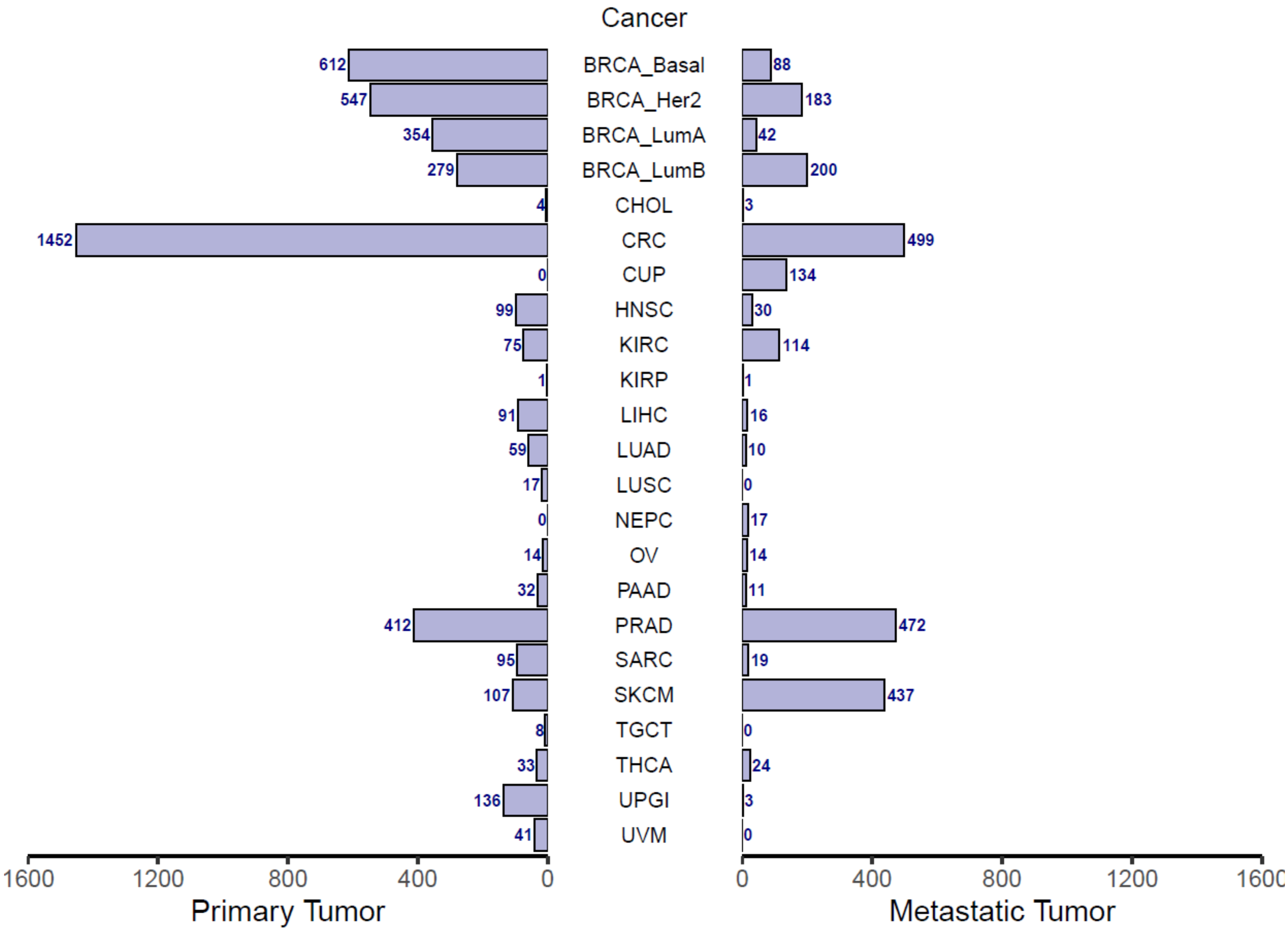

Figure S3: Microarray Sample Size
Sample sizes for each cancer type, separated into primary and metastatic tumors.

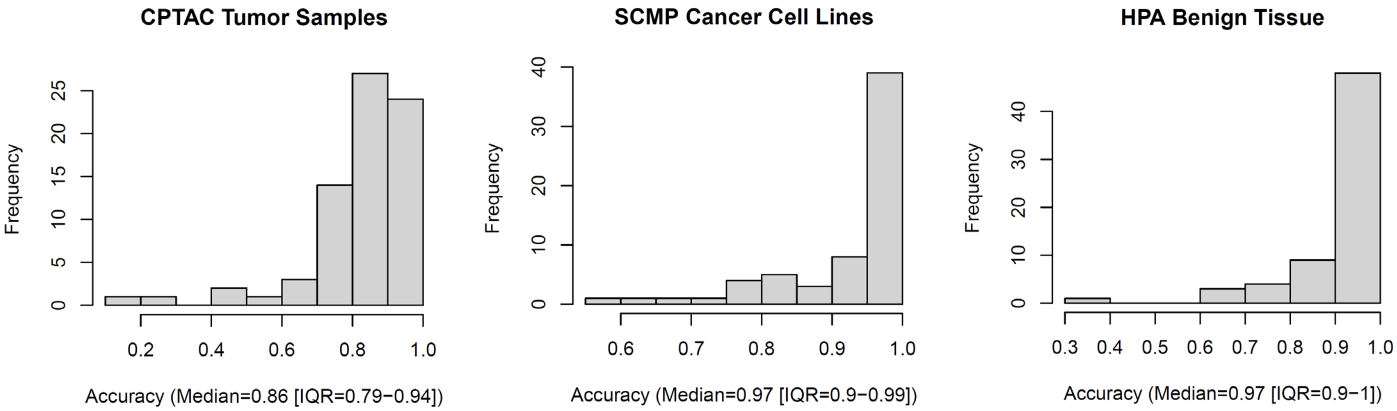

Figure S4: High Cell Surface Target RNA Expression Accurately Predicts High Protein Expression
RNA vs. protein expression in three datasets with matched RNA-seq and mass spectrometry: cancer tissue samples from the Clinical Proteomic Tumor Analysis Consortium (CPTAC), cancer cell lines from the Sanger Cell Model Passports (SCMP), and benign tissues from the Human Protein Atlas (HPA). We defined high expression of protein or RNA expression as being higher than 90% of housekeeping gene protein or RNA expression respectively in each sample, and histograms showing the distribution of accuracies across cell surface targets comparing protein and RNA are shown.

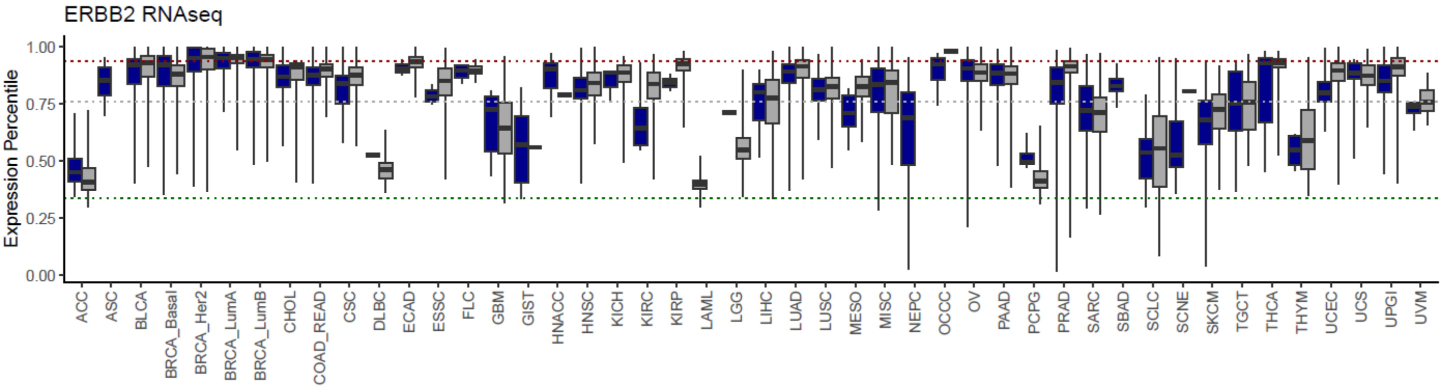
ERBB2 RNAseq
Expression Percentile
1.00
0.75
0.50
0.25
0.00
ACC ASC BLCA BRCA_Basal BRCA_Her2 BRCA_LumA BRCA_LumB CHOL COAD_READ CSC DLBC ECAD ESSC FLC GBM GIST HNACC HNSC KICH KIRC KIRP LAML LGG LIHC LUAD LUSC MESO MISC NEPC OCCC OV PAAD PCPG PRAD SARC SBAD SCLC SCNE SKCM TGCT THCA THYM UCEC UCS UPGI UVM

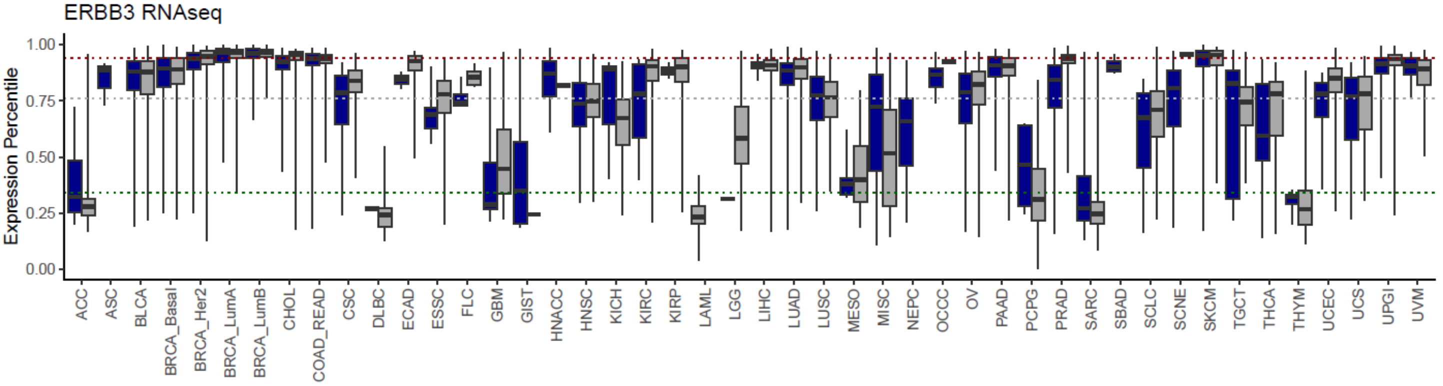
ERBB3 RNAseq
Expression Percentile
1.00
0.75
0.50
0.25
0.00
ACC ASC BLCA BRCA_Basal BRCA_Her2 BRCA_LumA BRCA_LumB CHOL COAD_READ CSC DLBC ECAD ESSC FLC GBM GIST HNACC HNSC KICH KIRC KIRP LAML LGG LIHC LUAD LUSC MESO MISC NEPC OCCC OV PAAD PCPG PRAD SARC SBAD SCLC SCNE SKCM TGCT THCA THYM UCEC UCS UPGI UVM

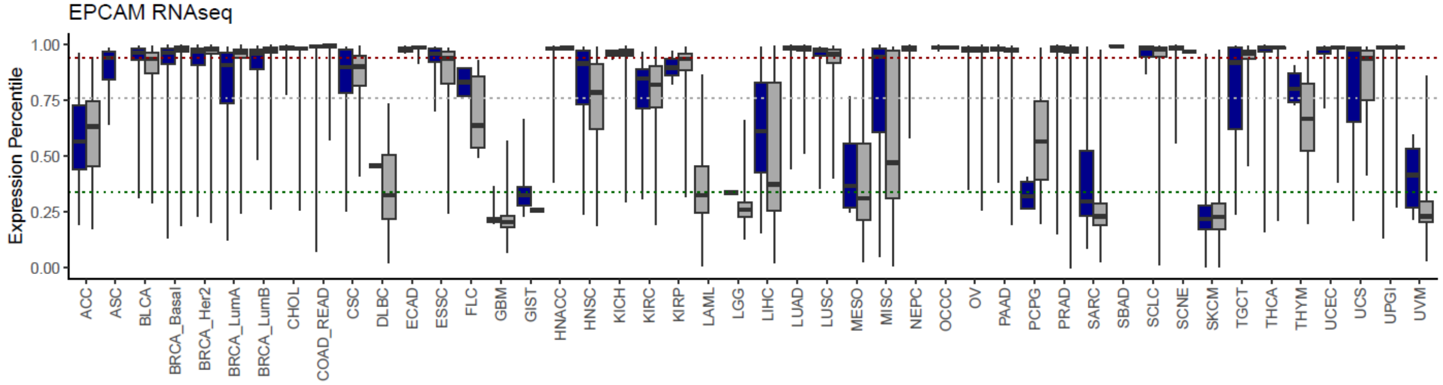
EPCAM RNAseq
Expression Percentile
1.00
0.75
0.50
0.25
0.00
ACC ASC BLCA BRCA_Basal BRCA_Her2 BRCA_LumA BRCA_LumB CHOL COAD_READ CSC DLBC ECAD ESSC FLC GBM GIST HNACC HNSC KICH KIRC KIRP LAML LGG LIHC LUAD LUSC MESO MISC NEPC OCCC OV PAAD PCPG PRAD SARC SBAD SCLC SCNE SKCM TGCT THCA THYM UCEC UCS UPGI UVM

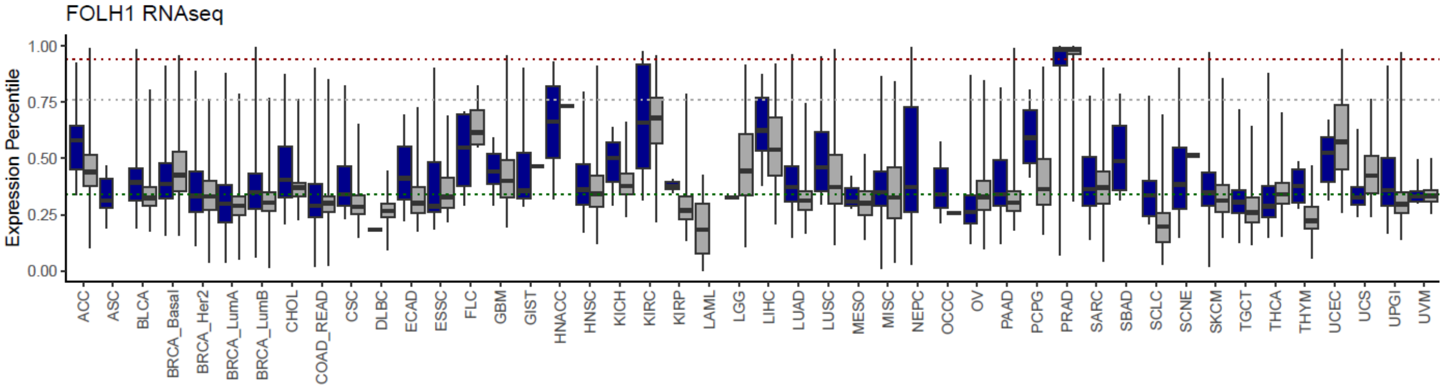
FOLH1 RNAseq
Expression Percentile
1.00
0.75
0.50
0.25
0.00
ACC ASC BLCA BRCA_Basal BRCA_Her2 BRCA_LumA BRCA_LumB CHOL COAD_READ CSC DLBC ECAD ESSC FLC GBM GIST HNACC HNSC KICH KIRC KIRP LAML LGG LIHC LUAD LUSC MESO MISC NEPC OCCC OV PAAD PCPG PRAD SARC SBAD SCLC SCNE SKCM TGCT THCA THYM UCEC UCS UPGI UVM

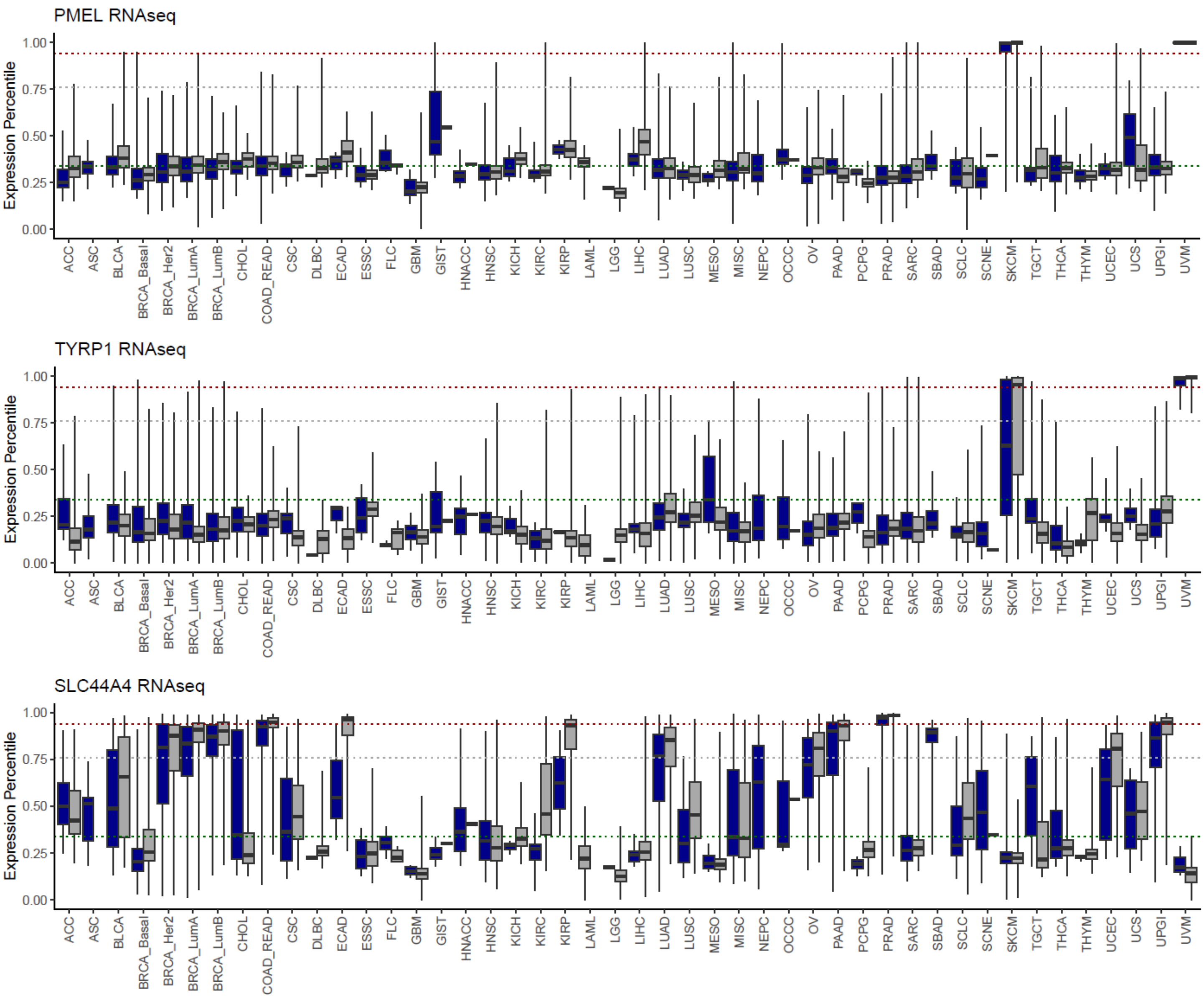

Figure S5: RNAseq Expression Consistent with Known Patterns for Cell Surface Targets

Boxplots show the metastatic (blue) and primary (gray) cancer expression in the RNAseq data for various cell surface targets. RNA expression levels are percentile rank normalized, ranging from 0 to 1. Green/gray/red dotted line: 1st/50th/90th percentile of housekeeping gene expression. We benchmarked the percentile expression data to housekeeping gene expression. The 94th, 76th, and 34th percentiles of overall gene expression represented a level ≥90%, ≥50%, and ≥1% of housekeeping genes

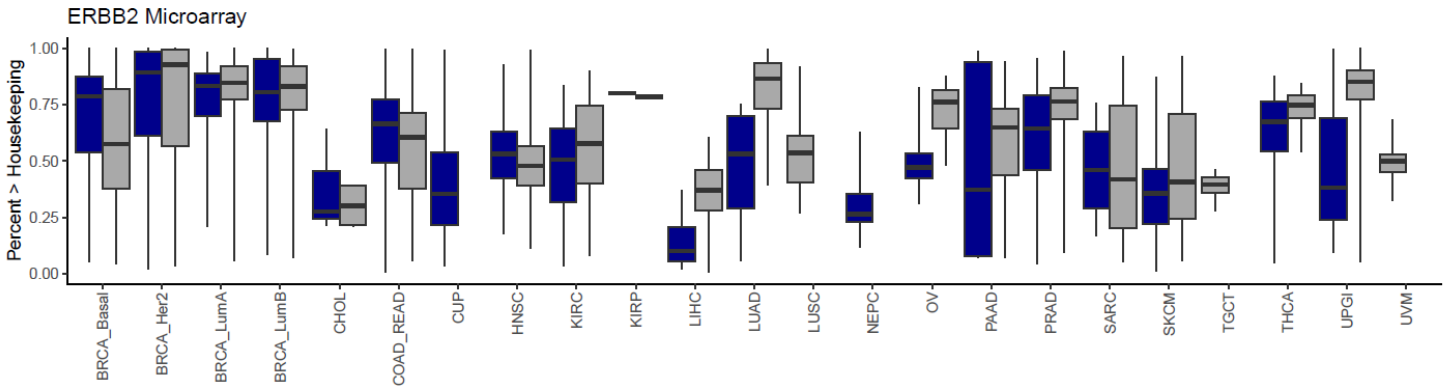
ERBB2 Microarray
Percent > Housekeeping
1.00
0.75
0.50
0.25
0.00
BRCA_Basal
BRCA_Her2
BRCA_LumA
BRCA_LumB
CHOL
COAD_READ
CUP
HNSC
KIRC
KIRP
LIHC
LUAD
LUSC
NEPC
OV
PAAD
PRAD
SARC
SKCM
TGCT
THCA
UPGI
UVM

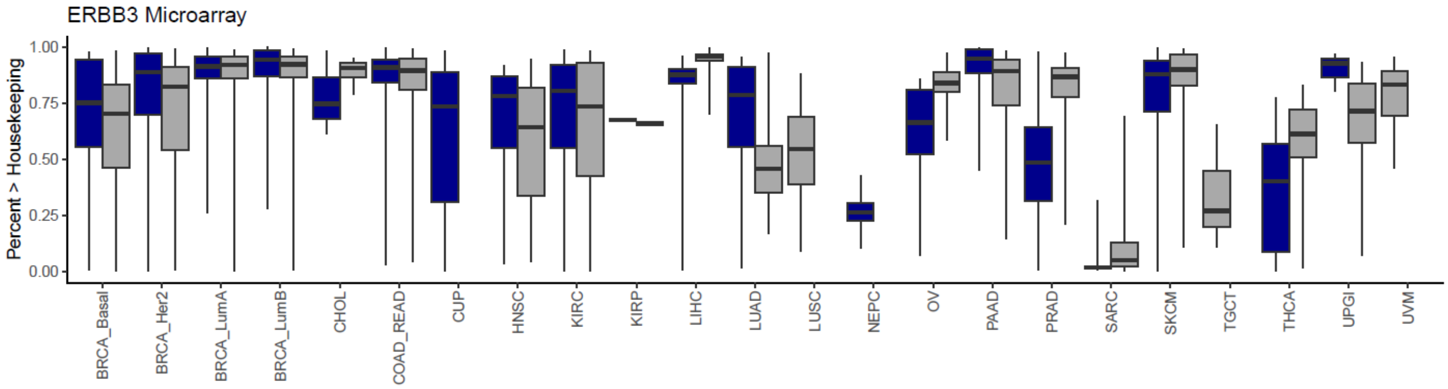
ERBB3 Microarray
Percent > Housekeeping
1.00
0.75
0.50
0.25
0.00
BRCA_Basal
BRCA_Her2
BRCA_LumA
BRCA_LumB
CHOL
COAD_READ
CUP
HNSC
KIRC
KIRP
LIHC
LUAD
LUSC
NEPC
OV
PAAD
PRAD
SARC
SKCM
TGCT
THCA
UPGI
UVM

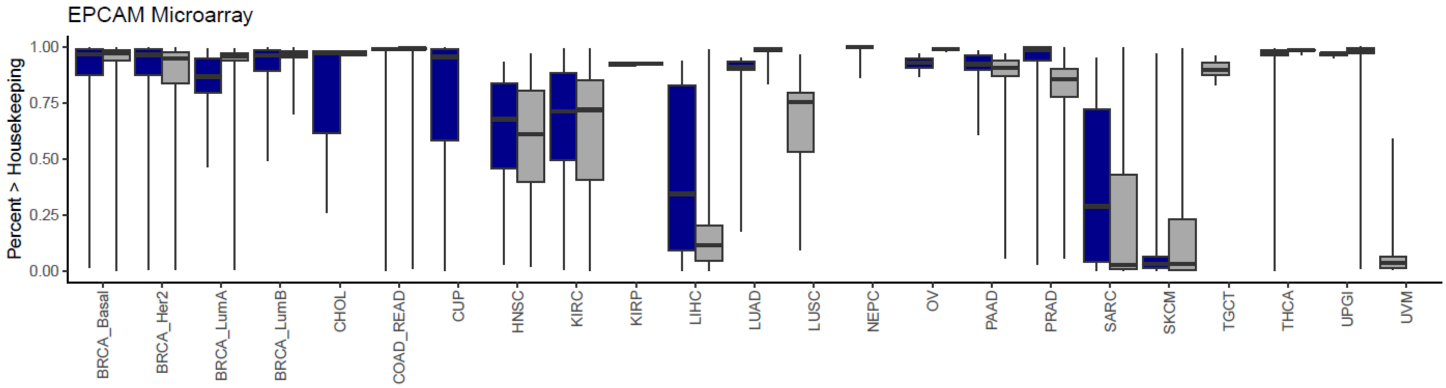
EPCAM Microarray
Percent > Housekeeping
1.00
0.75
0.50
0.25
0.00
BRCA_Basal
BRCA_Her2
BRCA_LumA
BRCA_LumB
CHOL
COAD_READ
CUP
HNSC
KIRC
KIRP
LIHC
LUAD
LUSC
NEPC
OV
PAAD
PRAD
SARC
SKCM
TGCT
THCA
UPGI
UVM

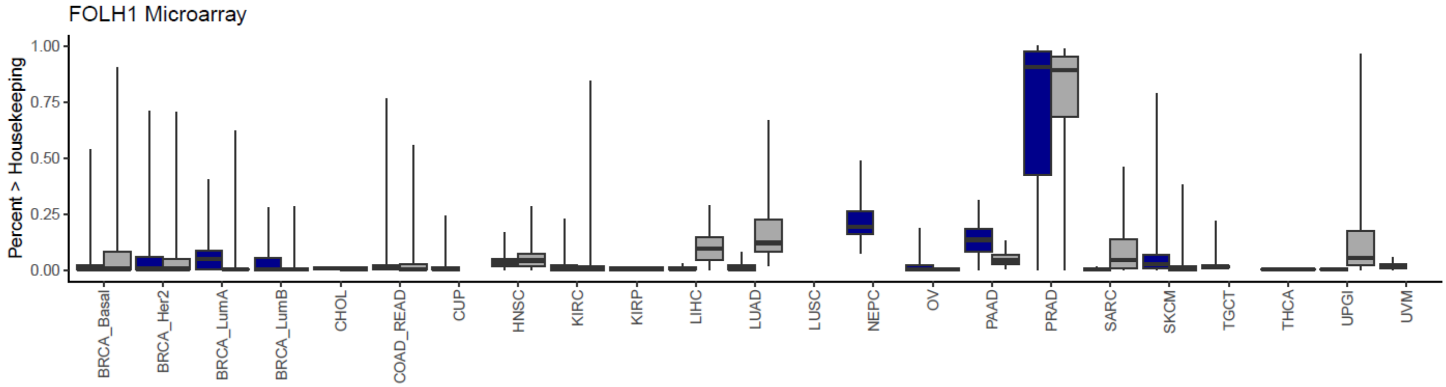
FOLH1 Microarray
Percent > Housekeeping
1.00
0.75
0.50
0.25
0.00
BRCA_Basal
BRCA_Her2
BRCA_LumA
BRCA_LumB
CHOL
COAD_READ
CUP
HNSC
KIRC
KIRP
LIHC
LUAD
LUSC
NEPC
OV
PAAD
PRAD
SARC
SKCM
TGCT
THCA
UPGI
UVM

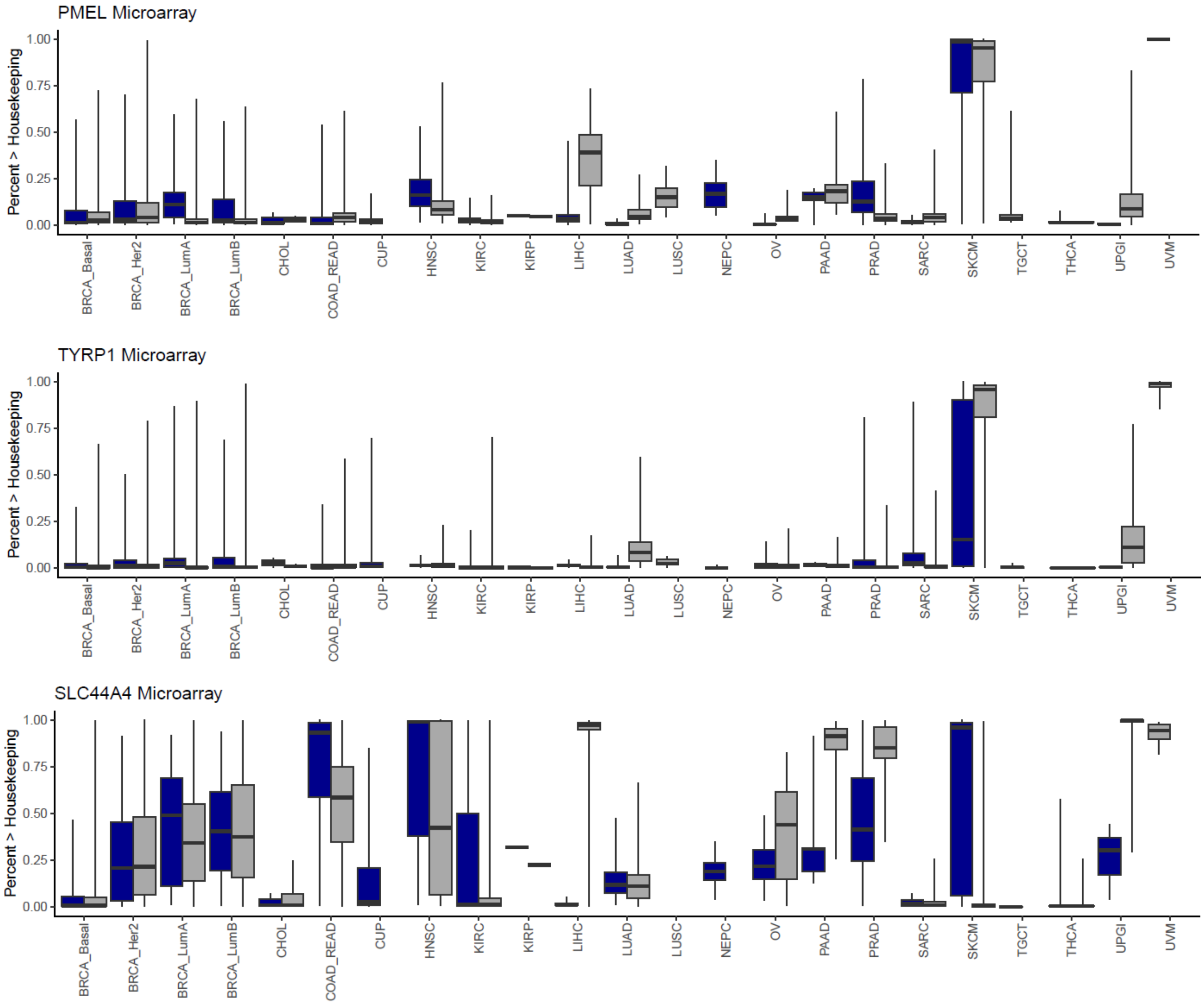

Figure S6: Microarray Expression Consistent with Known Patterns for Cell Surface Targets
Boxplots show the metastatic (blue) and primary (gray) cancer expression in the microarray data for various cell surface targets. Expression for each gene is normalized to be the fraction of housekeeping genes with lower expression (e.g. a gene expressed higher than 90% of housekeeping genes in a sample = 0.9).

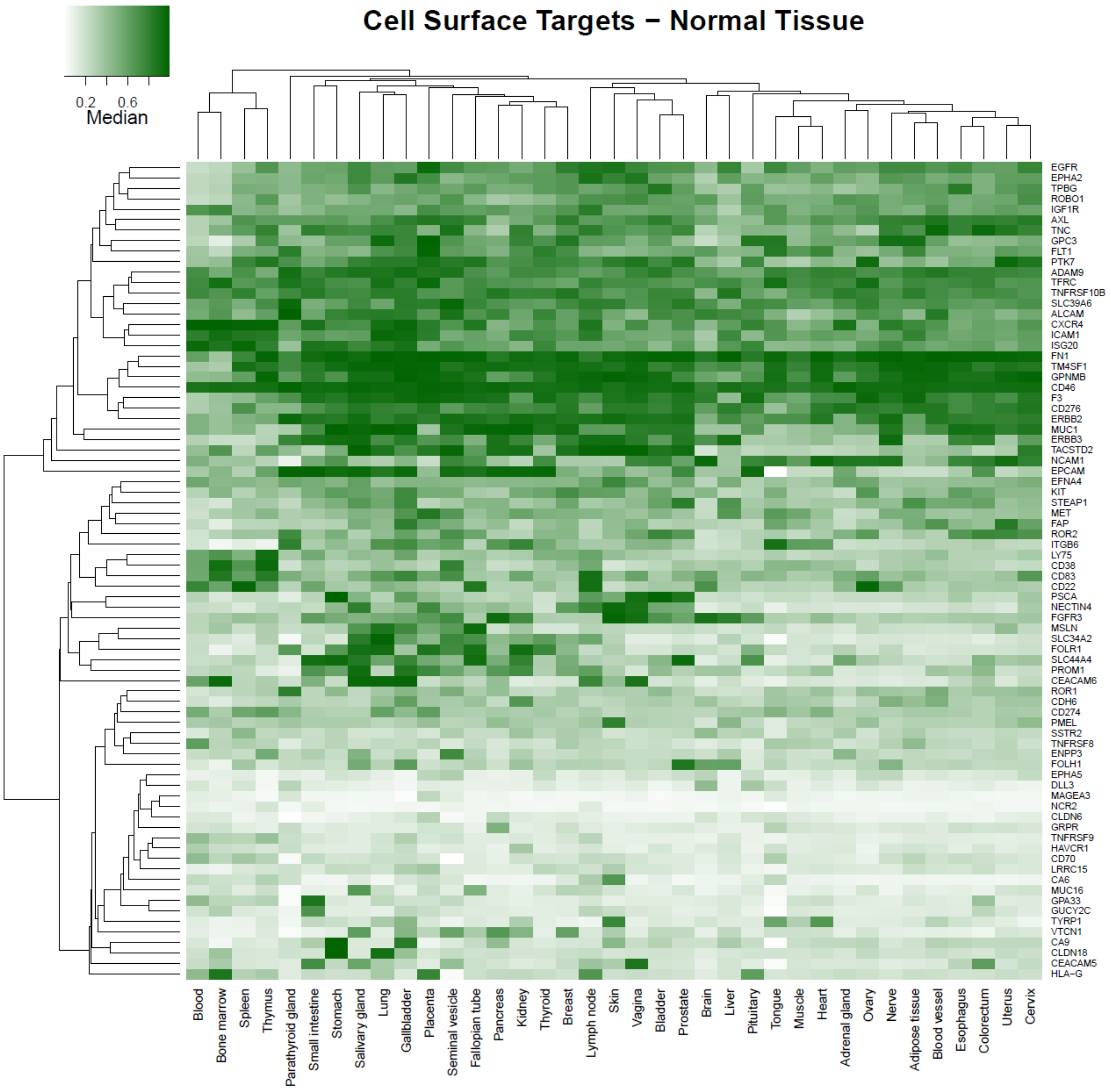

Figure S7: Normal Tissue Expression

Hierarchical clustering of median bulk RNA expression of 78 cell surface protein targets across 37 normal tissue types. RNA expression levels are percentile normalized, with values ranging from 0 to 1.

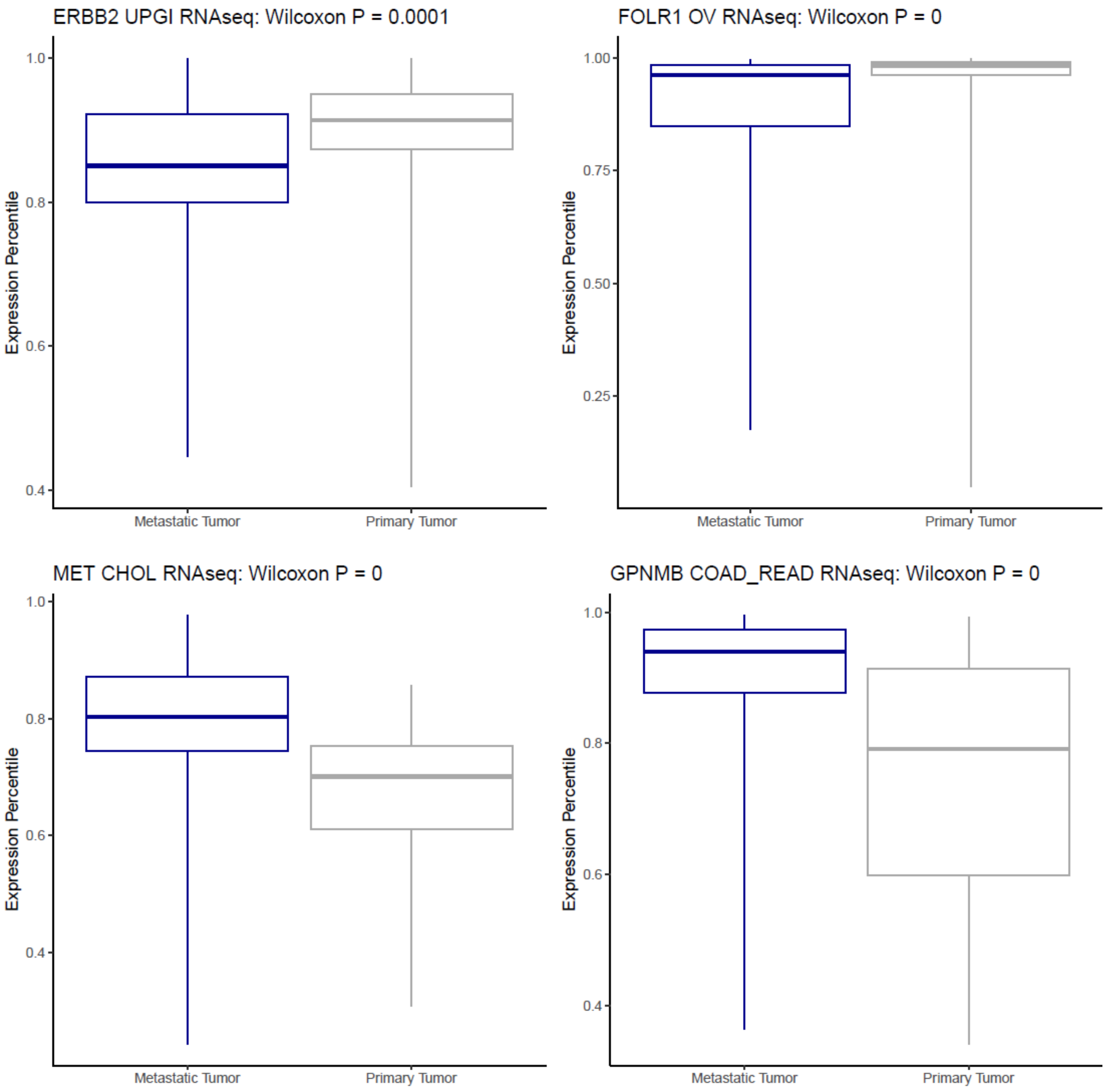

Figure S8: Examples of Primary vs. Metastatic Dichotomy
Boxplots show the metastatic (blue) and primary (gray) cancer expression in the RNAseq data for various cell surface targets. Two-sided Wilcoxon Rank-Sum test performed.

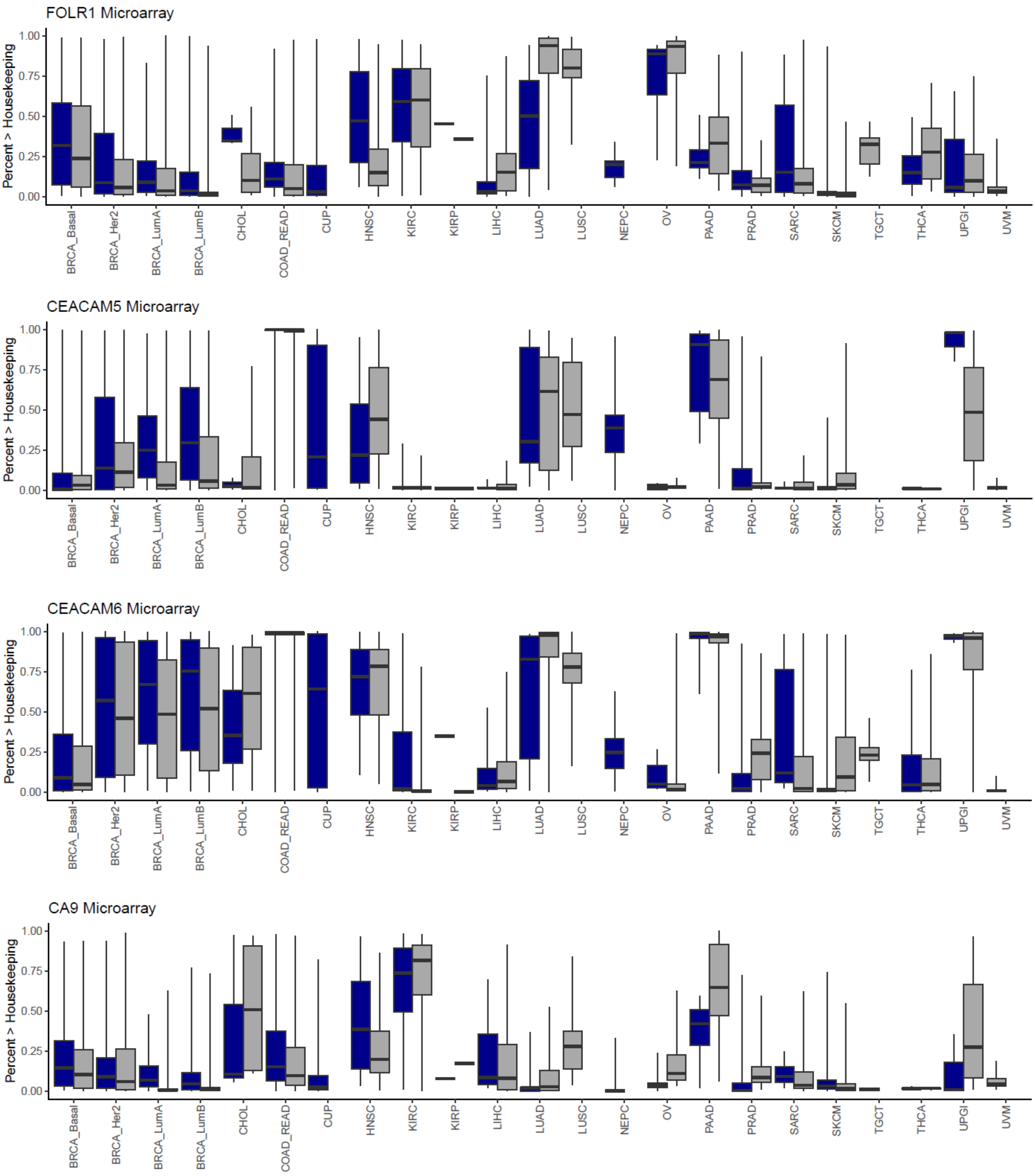
FOLR1 Microarray
CEACAM5 Microarray
CEACAM6 Microarray
CA9 Microarray
Percent > Housekeeping
0.00
0.25
0.50
0.75
1.00
BRCA_Basal
BRCA_Her2
BRCA_LumA
BRCA_LumB
CHOL
COAD_READ
CUP
HNSC
KIRC
KIRP
LIHC
LUAD
LUSC
NEPC
OV
PAAD
PRAD
SARC
SKCM
TGCT
THCA
UPGI
UVM

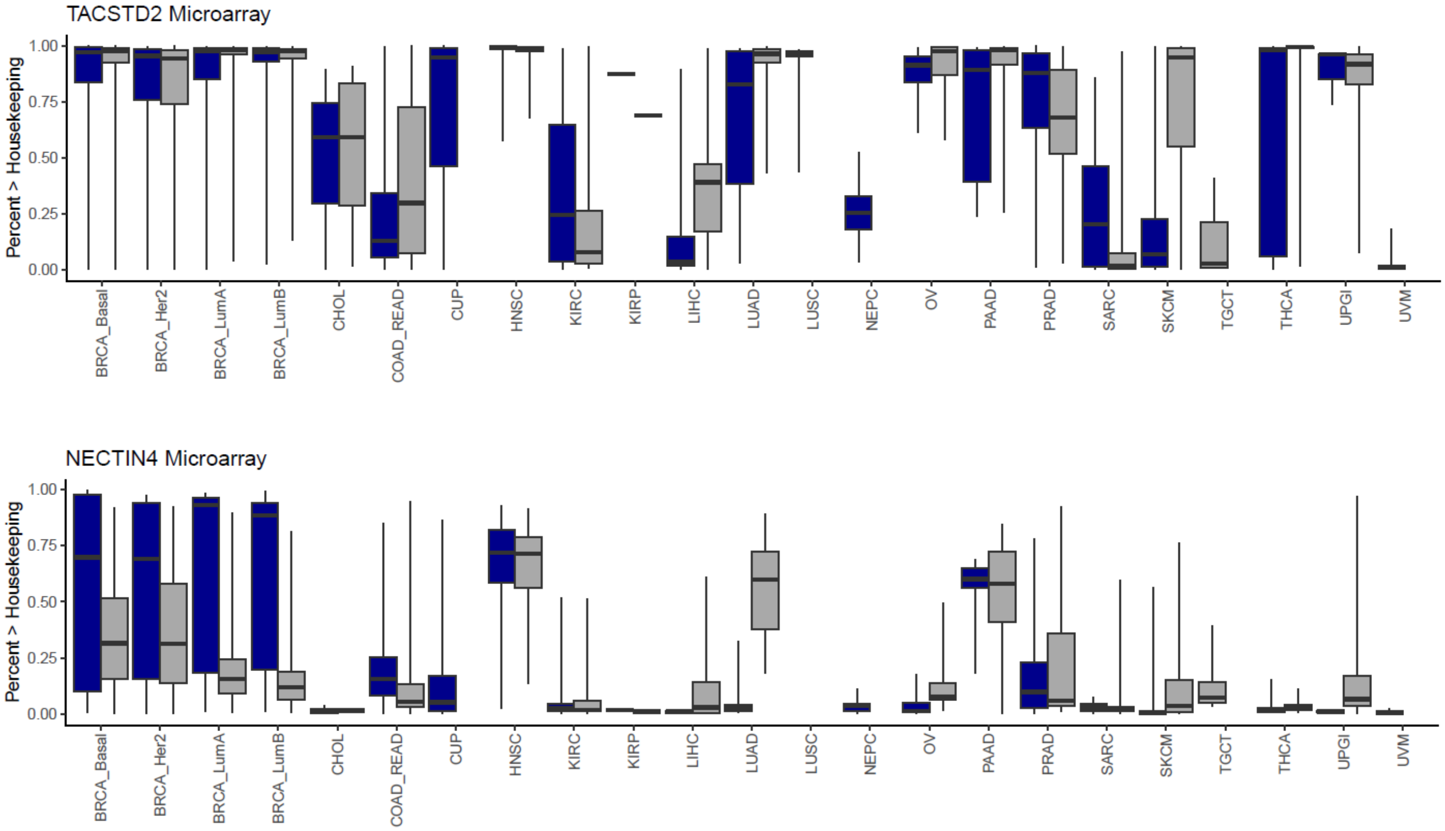

Figure S9: Drug Repositioning Candidates – Microarray
Boxplots show the metastatic (blue) and primary (gray) cancer expression in the microarray data for various cell surface targets. Expression for each gene is normalized to be the fraction of housekeeping genes with lower expression (e.g. a gene expressed higher than 90% of housekeeping genes in a sample = 0.9).

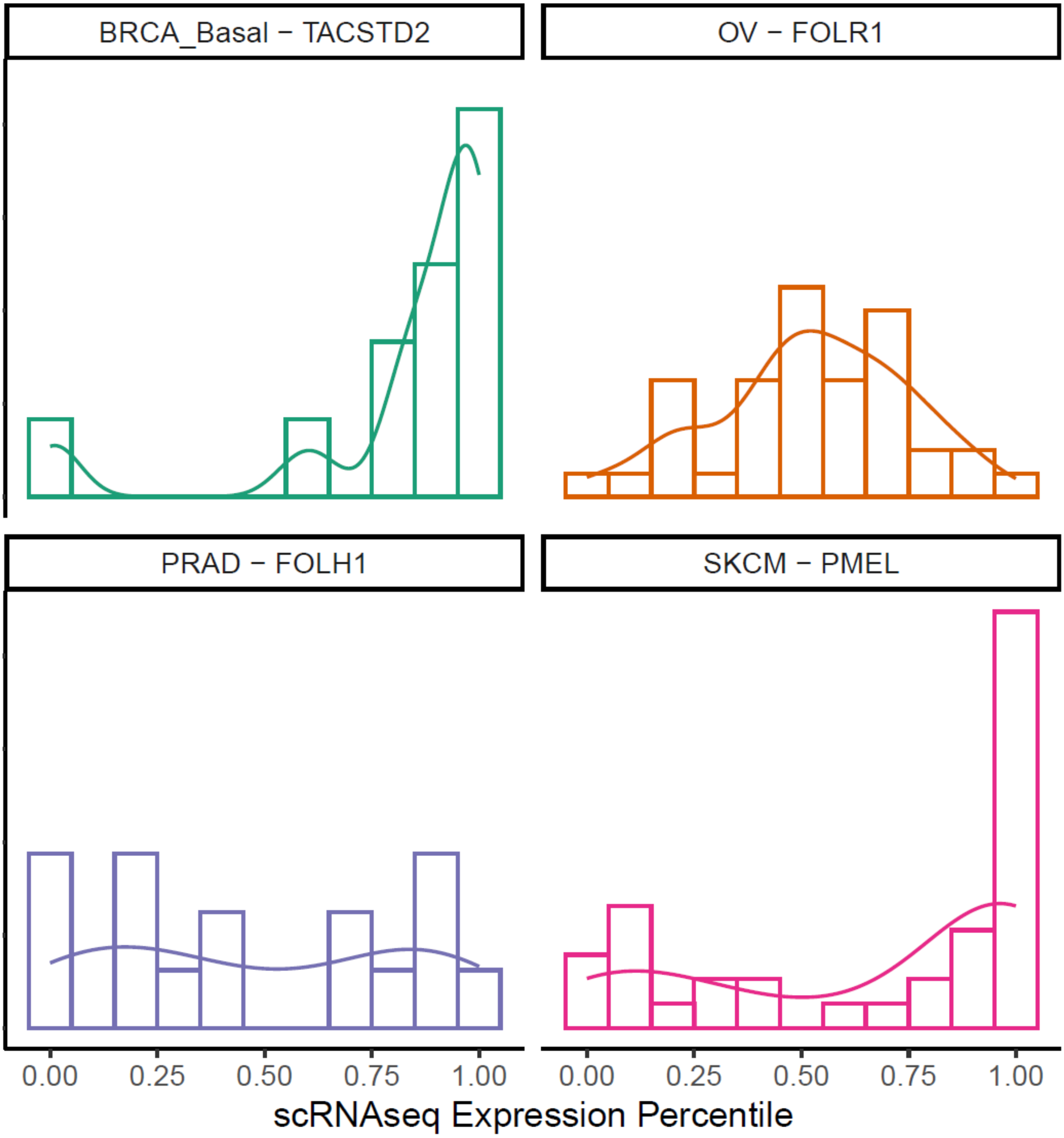

Figure S10: Single-cell RNAseq Distributions
Example distributions of the proportion of the tumor cells in each single-cell RNAseq sample that expresses each cell surface target, grouped by specific cell surface targets and tumor types.
